# Genetic analysis reveals Finnish *Formica fennica* populations do not form a separate genetic entity from *F. exsecta*

**DOI:** 10.7717/peerj.6013

**Published:** 2018-12-06

**Authors:** Sanja Maria Hakala, Perttu Seppä, Maria Heikkilä, Pekka Punttila, Jouni Sorvari, Heikki Helanterä

**Affiliations:** 1 Organismal and Evolutionary Biology Research Programme, Faculty of Biological and Environmental Sciences, University of Helsinki, Helsinki, Finland; 2 Tvärminne Zoological Station, University of Helsinki, Hanko, Finland; 3 Finnish Museum of Natural History, University of Helsinki, Helsinki, Finland; 4 Finnish Environment Institute, Helsinki, Finland; 5 Department of Environmental and Biological Sciences, University of Eastern Finland, Kuopio, Finland; 6 Ecology and genetics research unit, University of Oulu, Oulu, Finland

**Keywords:** Species identification, Species delimitation, Hymenoptera, *Coptoformica*, Microsatellites, Barcoding

## Abstract

*Coptoformica* Müller, 1923 is a subgenus of *Formica* Linnaeus, 1758 that consists of c. a dozen species of ants that typically inhabit open grassy habitats and build small nest mounds. The most recent addition to the group is *Formica fennica* Seifert, 2000. The description was based on morphological characters, but the species status has not been confirmed by molecular methods. In this study, we use thirteen DNA microsatellite markers and a partial mitochondrial COI gene sequence to assess the species status of *F. fennica*, by comparing the genetic variation among samples identified as *F. fennica* and six other boreal *Formica (Coptoformica)* species. Most of the species studied form separate, discontinuous clusters in phylogenetic and spatial analyses with only little intraspecific genetic variation. However, both nuclear and mitochondrial markers fail to separate the species pair *F. exsecta* Nylander, 1846 and *F. fennica* despite established morphological differences. The genetic variation within the *F. exsecta/fennica* group is extensive, but reflects spatial rather than morphological differences. Finnish *F. fennica* populations studied so far should not be considered a separate species, but merely a morph of *F. exsecta*.

## Introduction

Species is one of the fundamental units in biology, but it is also one that is very hard to define. There are many different and sometimes contrasting species concepts, which can be summarized with an unified species concept: a species is a separately evolving metapopulation lineage (De Queiroz, 2007). The practical delimitation of species can depend on several features (De Queiroz, 2007), and it can be difficult due to some lineages lacking easily distinguishable features (Bickford et al., 2007). In particular, taxa that rely on chemical communication instead of visual cues can be very cryptic to human observers (Mayr, 1963), which is a plausible explanation for the high amount of cryptic diversity found in ants (Seifert, 2009). Also, hybridization between recently diverged lineages is common and further complicates species delimitation (Abbott et al., 2013)—also in ants (Seifert, 1999). To some extent, inferring the boundaries between species has always been and will always be a matter of agreement and a subject to debate.

Nonetheless, assessing where the species boundaries are is crucial for biologist, and all fields of biology rely on species delimiting done by taxonomists, and the species identification criteria they provide. Using the most up-to-date knowledge is especially important in ecology and population biology, where the behavior or genetics of multiple populations is studied simultaneously. The conclusions of these studies depend on the correct species identifications. Bortolus (2008) argues that mistakes in taxonomy in ecological studies can have major cascading errors in our understanding of nature. Ecological studies and descriptions of biodiversity are also the basis on which conservation decisions are made, and thus correct species identification should be a main concern (Bortolus, 2008; Pante et al., 2015).

Ants in the genus *Formica* Linnaeus, 1758 have been widely studied due to their social behavior and ecological importance. Species delimitation and identification in this taxon is difficult because some of the species are relatively young (Goropashnaya et al., 2012). Hybridization is common between closely-related species, and the hybrids are often fertile (Czechowski, 1993; Seifert, 1999; Goropashnaya, Fedorov & Pamilo, 2004; Korczyńska et al., 2010; Kulmuni, Seifert & Pamilo, 2010). Especially morphological species identification of ants and other social insects has a major practical difficulty that is unique to these taxa (Ward, 2010): in many studies species identification is done from worker samples alone, since sampling sexual castes, i.e., the reproductive queens and males, is more difficult. Compared to sexual castes, workers have less morphological variation among species and often more variation within species, which makes species identification with morphological attributes especially difficult (Ward, 2010).

*Coptoformica* Müller, 1923, is a subgenus of *Formica*. Ants in this subgenus live in open habitats and build small nest mounds of grass, typically 20–40 cm high in some of the species, and very low heaps in some of the species (Seifert, 2000; Punttila & Kilpeläinen, 2009) with a basal area varying greatly (Sorvari, 2009). They chop nest material into smaller pieces with their strong mandibles and jaw muscles that extend into the occipital corners of their heads, which gives them their distinctive heart-shaped heads (Seifert, 2000). The group includes c. 12 species in the Palaearctic (Seifert, 2000; Schultz & Seifert, 2007). Since the *Coptoformica* subgenus has several rare species, and their preferred habitats, such as meadows and mires, belong to the most threatened habitat types in Finland (Kontula & Raunio, 2009), they are a candidate group for future conservation efforts. According to National Red Lists (2018) several species of the group are threatened at varying levels in different European countries, and many of them have declining populations (Seifert, 2000). At the moment in Finland, only one of the species, *Formica suecica* Adlerz, 1902 is classified as Near Threatened (IUCN Red List Category NT) due to the overgrowing of their preferred habitats, whereas the rest of the five species occurring in Finland are classified as Least Concern (IUCN Red List Category LC) (Rassi et al., 2010).

The most recently described species is *Formica fennica* Seifert, 2000. The description is based on morphological features of samples collected from three locations in Finland and one in the Caucasus, Georgia, with one queen sample from Finland (Kitee) denoted as the holotype (Seifert, 2000). Since then, the species has been identified from several other locations in Finland, predominantly from mires in the northern parts of the country (Punttila & Kilpeläinen, 2009), from one location in Norway, also from “wet conditions” (Suvák, 2013), and from another location in the Caucasus, Azerbaijan (Schultz & Seifert, 2007). Based on morphology, *F. fennica* and Central Asian *F. manchu* Wheeler, 1929 are considered to be sister species (Seifert, 2000), but locally in Finland, *F. fennica* is both morphologically and ecologically very similar to *F. exsecta* Nylander, 1846. *Formica exsecta* is the most widely distributed species of the subgenus (Schultz & Seifert, 2007), and it is very variable both morphologically and ecologically (Seifert, 2000). According to Seifert (2000), *F. exsecta* and *F. fennica* are separated from each other by the distribution of standing setae on the gastral terga and clypeus, and by the number of semi-erect setae on the craniad profile of forecoxae. Since there is a lot of variation in each of these characteristics in both species, nest samples with multiple workers are needed to calculate the averages of the characteristics for the separation of the species (Seifert, 2000).

The identification of *F. fennica* is further complicated by the existence of a pilosity-reduced form of *F. exsecta*, that was originally described as a separate species called *Formica rubens* Forel, 1874. Not much is currently known of this morph. Based on the original description, *F. rubens* is larger and more brightly and evenly red than *F. exsecta* (Forel, 1874). According to current understanding, intraspecific color morphs are very common in ants, as is size variation, and usually these kinds of characters are not adequate for species identification (Seifert, 2009). *Formica rubens* was recently synonymized with *F. exsecta* based on the examination of four individuals of the type series collected from Switzerland, because all morphological characters measured were within the range of *F. exsecta* (Seifert, 2000). However, after the synonymization, Ødegaard (2013) stated: “*F. (C.) rubens* is interpreted as a mutant conspecific with *F. (C.) exsecta* (Bernhard Seifert in litt.), but it is not impossible that *F. rubens* may turn out to be a good species in the future.” Ødegaard (2013) recommended using extreme caution when identifying *F. fennica*. In Ødegaard’s (2013) data from *Coptoformica* colonies from mires in Hedmark, Norway, several colony samples fit the description of *F. fennica* based “on the presence of microhairs on the eyes combined with lack of setae on T1 and T2 and partly T3, and 0–3 setae on the front of the fore coxae”, but these were identified to represent the setae-reduced *rubens* mutant of *F. exsecta*, even though the details of the identification are not mentioned. Similarly, the Finnish mire samples of *Formica fennica* studied by Punttila & Kilpeläinen (2009) were identified using the same key (Seifert, 2000), but it cannot be ruled out that they represent another case of the *rubens* morph.

The close resemblance to *F. exsecta* and *F. fennica*, and the lack of molecular verification of the species status of *F. fennica* created the need for this study. Using only morphological methods for species identification is risky in the presence of intermediate individuals, and makes further ecological studies on these two species very difficult. Molecular methods have proven to be especially valuable when working with morphologically conserved species groups: the number of described cryptic species has increased exponentially after the introduction of polymerase chain reaction (PCR) and the molecular methods it enables (Bickford et al., 2007). Conversely, also two morphs of a same species can be erroneously described as two separate species, which are later synonymized after more thorough examination. This is not a trivial phenomenon: A Web of Science search (January 2018) with the search query “synonymiz* OR synonymis*” yields 3,658 articles labelled with some ecological field (zoology, entomology, plant sciences, evolutionary biology, biodiversity conservation, marine freshwater biology, ecology, mycology, parasitology, microbiology, limnology, ornithology). Thus, confirming the taxonomy of studied taxa with molecular methods is very advisable.

In this work we evaluate the existence of *F. fennica* as a separate species using molecular methods, and investigate its position in *Formica* phylogeny (Goropashnaya et al., 2012). The goal of this study is to test whether *F. fennica* is a separately evolving lineage in the same extent as the other species of the subgenus. Given that we use genetic data as our sole line of evidence, our approach is consistent with the biological species concept that emphasizes reproductive isolation and the lack of gene flow as the most important species delimiting properties (Mayr, 1942). The hypothesis is that all seven morphologically identified *Coptoformica* species included in this study are recovered as separate lineages also in analyses based on molecular methods. Further, we test the hypothesis of *F. fennica* being a sister species of *F. manchu* (Seifert, 2000) among the limited number of species used in this study*.* The aim of the sampling scheme is to investigate the species status of *F. fennica* in Finland, in the currently known core area of its distribution, leaving other biogeographical areas outside the scope of this study.

## Materials and Methods

### Sampling and species identification

**Figure 1:**
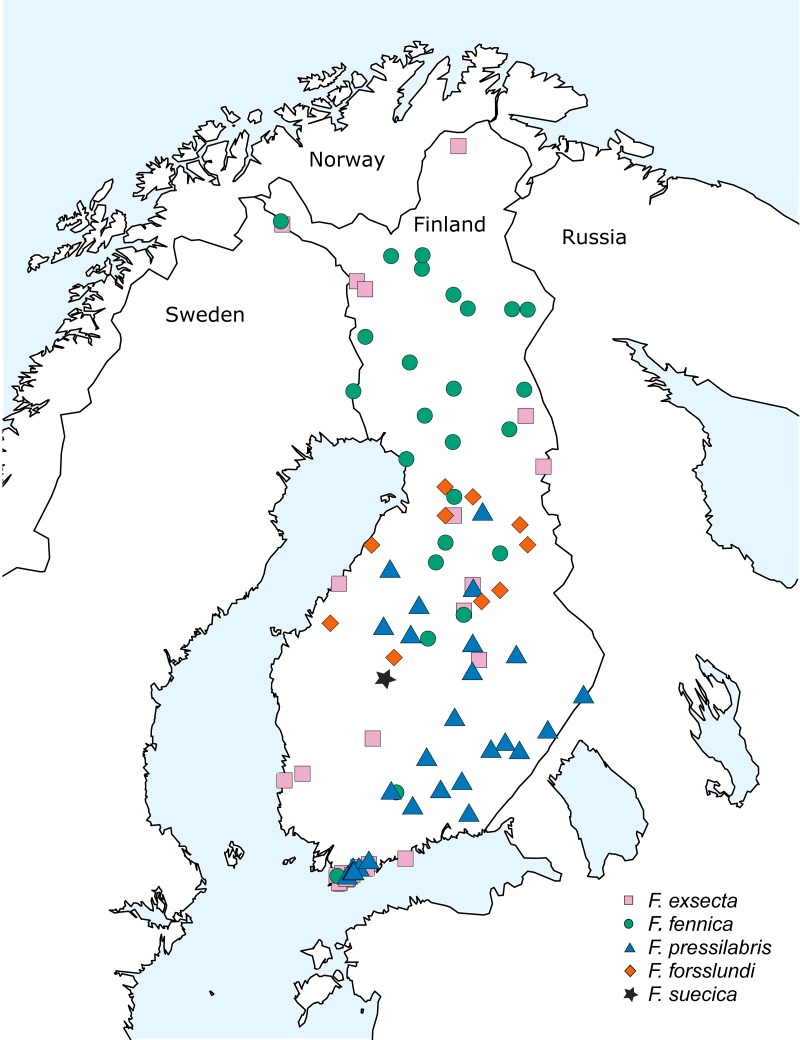
Sampling locations in Finland.

The bulk of samples used in this study were collected during the 10th Finnish National Forest Inventory (NFI) carried out by the Natural Resources Institute Finland (earlier Finnish Forest Research Institute) during the years 2005–2008, also used by Punttila & Kilpeläinen (2009). This dataset was supplemented with additional samples from various areas of Finland, collected during the years 2008–2015 (Fig. 1; Table S1). The study area covers over 1,100 kilometers in south-north direction, reaching from the hemiboreal zone to the northern border of the northern boreal zone. Samples of two additional species from eastern Siberia, *Formica pisarskii* Dlussky, 1964 and *F. manchu* were also included. These samples were originally collected for another study (Goropashnaya et al., 2012) and the original morphological identifications were done by B. Seifert (P. Pamilo in litt.).

The samples collected in Finland included usually 15–20 individuals per nest, but in rare cases—when the amount of active workers was low due to bad weather or to the weakened condition of the nest population—this amount was not achieved. The ants were identified with the key of Seifert (2000) using sample averages of the critical characteristics based on five or more worker individuals. When identifying *F. fennica* from less hairy samples of *F. exsecta*, 10–20 workers were inspected. When the sample contained less workers, all the available individuals were checked. All individuals identified as *F. fennica* using this key are hereafter referred to with this name, with the understanding that identifications as the *rubens* morph of *F. exsecta* might in some cases be more accurate (Ødegaard, 2013). In total, the Finnish dataset includes all the five *Coptoformica* species known to occur in Finland: *F. fennica* (33 nests from 26 locations), *F. exsecta* (38/27), *F. pressilabris* Nylander, 1846 (42/29*), F. forsslundi* Lohmander, 1949 (13/10), *F. suecica* (2/1). The geographic distributions of the seven study species are given in Table 1.

**Table 1:** Geographic distributions of the study species in the Palaearctic Region, reproduced from Schultz & Seifert (2007).

**Species**	**Distribution in Europe**	**Distribution in Asia**
*F. exsecta*	Temperate to boreal, planar to subalpine and submeridional-subalpine	Oreo-Turanian and Tibetan to boreal, montane to subalpine
*F. fennica*	South boreal and Caucasian-montane	?
*F. forsslundi*	Temperate to boreal, planar to submontane	Tibetan to Central-Siberian-Daurian
*F. manchu*	−	Tibetan to Central-Siberian-Daurian
*F. pisarskii*	−	Mongolian to Central-Siberian-Daurian
*F. pressilabris*	Temperate to south boreal, planar to subalpine	Tibetan to Central-Siberian-Daurian and East Manshurian, montane to subalpine
*F. suecica*	North temperate to boreal, in the Alps montane to subalpine	−/?

**Notes.**

– not present

? not known

One *F. fennica* population included in this study (samples: FF_178 —FF_181) was one of the three Finnish populations used in the original species description (Seifert, 2000) and sampled by the same researcher (J. Sorvari) 12 years after the original sampling. Between these two sampling times the continuity of the population had been monitored yearly by J. Sorvari.

### Molecular methods and data analysis

DNA of two individuals per nest was extracted using Chelex^©^ (Biorad, Hercules, CA, USA) extraction protocol or NucleoSpin^®^ Tissue Kit by Macherey-Nagel. Same individuals were used for both microsatellite genotyping and DNA barcoding. As NFI samples were not originally collected for a genetic study, their storage conditions had not been optimal, resulting often in poor quality DNA, and several samples could not be sequenced successfully. Most of the poor quality samples were *F. fennica* samples, likely because they had previously been most intensively handled for the morphological identification. However, shorter microsatellite fragments could be amplified for almost all of these samples too. The *F. fennica* samples from the population originally used for species description are of good quality and were sequenced without problems. Detailed protocols for both DNA microsatellite methods and mtDNA sequencing together with a table of primer information are given in Table S2.

#### DNA microsatellite genotyping

Two individuals from each nest were genotyped to assess nuclear genetic variation within and between species, and to confirm the morphology-based species delimitations and identifications. Thirteen DNA microsatellite markers (Chapuisat, 1996; Gyllenstrand, Gertsch & Pamilo, 2002; Trontti, Tay & Sundström, 2003; Hasegawa & Imai, 2004) were used in four multiplexes with the Type-it Microsatellite PCR Kit (QIAGEN, Valencia, CA, USA) according to the manufacturer’s instructions. PCR products were analyzed with a 3730 ABI 3730 DNA analyzer (Applied Biosystems, Foster City, CA, USA) and alleles were scored using GeneMapper 5.0. (Applied Biosystems). DNA microsatellite data were analyzed with Genalex 6.502 (Peakall & Smouse, 2006; Peakall & Smouse, 2012), and R packages Hierfstat (Goudet, 2005) and Adegenet (Jombart, 2008). Six samples with more than 50% missing microsatellite data were omitted from further analyses.

Genetic variation at DNA microsatellite loci was described for all species with more than two sampling localities. For these species, pairwise *F*_ST_ values from the allelic distance matrix, and Nei’s standard genetic distances D (Nei, 1972) were calculated to assess genetic differentiation between species. When the genetic differentiation between *F. exsecta* and *F. fennica* was found to be minor, the correlation of linear genetic distances and log-transformed geographical distances within *F. exsecta/fennica* subset was investigated with a maximum-likelihood population-effects (MLPE) model with Residual maximum likelihood (REML) estimation (Clarke, Rothery & Raybould, 2002; Van Strien, Keller & Holderegger, 2012) with the R package ‘lme4’ (Bates et al., 2015).

Separation of the species based on nuclear genetic variation was analyzed at the individual level with mixture analysis using model-based Bayesian clustering with software Baps 6.0 (Corander, Waldmann & Sillanpää, 2003; Corander, Marttinen & Mäntyniemi, 2006). Only one individual per nest was used to eliminate the possible effect of nest structure in the analysis, as previously done by Seppä et al. (2011). The software was allowed to find the most probable number of clusters with repeated runs using different upper limits for the cluster number (first 5 times K7—K20, and thereafter 20 times K11—K16). The hypothesis was that each morphologically identified species would form separate clusters. After the initial analysis revealed that several *F. exsecta* and *F. fennica* samples cluster together, the same procedure was repeated using only *F. exsecta* and *F. fennica* samples, to assess if there is finer scale clustering within this group (first 5 times K2—K25, and thereafter 20 times K18—K24).

Similar analyses were run also with software Structure (Pritchard, Stephens & Donnelly, 2000). However, the mathematical model used by Structure does not deal well with unbalanced sampling and low sample sizes (Kalinowski, 2011; Puechmaille, 2016), which is apparent in our data. Thus, although the overall results for the focal species are similar with both Baps and Structure, Baps was deemed to be more suitable with our sampling patterns. Therefore only the Baps results are discussed further.

Discriminant analysis of principal components, DAPC (Jombart et al., 2010) was done for the whole microsatellite dataset to assess if morphologically different samples would also form discontinuous clusters based on nuclear genetic variation. Cross-validation for the optimal number of principal components (PCs) was carried out as instructed by the developers, and based on the highest mean predictive success and lowest root mean squared error, 24 principal components (of the total 126) were included in the final DAPC. Missing data were substituted with the mean allele frequencies. The analysis was repeated with only *F. exsecta* and *F. fennica* samples in order to check how well the optimal model for this subset of data is able to separate the two species. Based on cross validation, 63 PCs (of the total 123) should be included for the analysis to achieve the highest accuracy and lowest error.

#### DNA barcoding

Part of the gene Cytochrome c oxidase subunit I (COI) was amplified for 85 samples from different nests to assess mitochondrial variation among the subgenus. All *F. fennica* samples that could be sequenced successfully were included in the analysis (24 samples), together with a geographically representative subset of samples from other species (leaving out samples collected from the same or nearby locations): *F. exsecta* (29), *F. pressilabris* (18), *F. forsslundi* (10), *F. suecica* (2), *F. manchu* (1), *F. pisarskii* (1) (details given in S1). PCR primers designed by Seppä et al. (2011) were used with the Phusion PCR kit (Finnzymes) according to manufacturer’s instructions. Amplification products were purified and sequenced in the Institute of Biotechnology of the University of Helsinki using the aforementioned primers.

The obtained 525 base-pair sequences were assembled and aligned with Geneious 8.1.7 (Biomatters) with Muscle alignment (Edgar, 2004). Sequence divergences as numbers and percentages of differing nucleotides were calculated for all pairs of haplotypes. Maximum likelihood analyses of the aligned barcode regions were performed using the program RAxML v8 (Stamatakis, 2014). The analyses were run in CIPRES (Miller, Pfeiffer & Schwartz, 2010) with the GTR model, and partitioned by codon position. Bootstrap support values were evaluated with 1,000 bootstrap replicates of the data and plotted onto the best scoring tree with Figtree (2018). Of the 85 *Coptoformica* sequences, 13 representing all the different haplotypes were included in the ingroup. An additional sequence of *F. exsecta* collected in Finland was obtained from GenBank (AB103364.1). The analyses included three species as outgroup: *Formica (Serviformica) lemani* Bondroit, 1917, *Formica (Formica s. str.) truncorum* Fabricius, 1804, and *Formica (Formica s. str.) pratensis* Retzius, 1783. The molecular data for these taxa were obtained from GenBank (AB019425.1, AB010929.1 and AB103363.1, respectively).

Six of the 24 *F. fennica* samples (290, 294, 296, 304, 310, 312) in the ingroup representing three different haplotypes were excluded from the final analysis because they placed with species in other subgenenera in the phylogeny. The risk of this result being due to contamination or an error in the sequencing of poor quality DNA, or due to nuclear copies of mitochondrial DNA, was considered too high. However, these samples did not stand out from other samples in the microsatellite dataset, and were therefore not excluded from microsatellite analyses, although two did have too much missing data and were excluded for this reason. The full phylogeny and haplotype distance table with all 85 sequences is presented in S4. The phylogeny of the 14 ingroup taxa representing the haplotypes of the remaining 79 sequences and the additional *F. exsecta* obtained from GenBank is hereafter presented and discussed.

## Results

The variation in the microsatellite markers is described in Table S3 . The amount of missing data is 2.16% for the whole microsatellite dataset of two individuals per nest, and 1.26% for the dataset of a single individual per nest.

The pairwise *F*_ST_ values (Table 2) between different species are generally much higher (0.15–0.20), than the values between *F. exsecta* and *F. fennica* (0.03). All *F*_ST_ values are significant (*p* < 0.001). Nei’s D (Table 2) show the same pattern with higher values between other species pairs (0.72–0.87) and lower values between *F. exsecta* and *F. fennica* (0.11). Among *F. exsecta/fennica* samples, the pairwise genetic distances are explained by geographical distance (MLPE: *β* = 0.34, *SE* = 0.02, *P* < 0.0001).

**Table 2:** Genetic differentiation between species. Below diagonal: pairwise FST values (*p* < 0.001 for all). Above diagonal: pairwise Nei’s genetic distance (D).

****	**Fe**	**Ff**	**Ffo**	**Fp**
Fe		0.110	0.872	0.799
Ff	0.025		0.788	0.748
Ffo	0.204	0.182		0.719
Fp	0.161	0.146	0.190	

**Notes.**

Fe *F. exsecta* Ff *F. fennica* Ffo *F. forsslundi* Fp *F. pressilabris*

**Figure 2:**
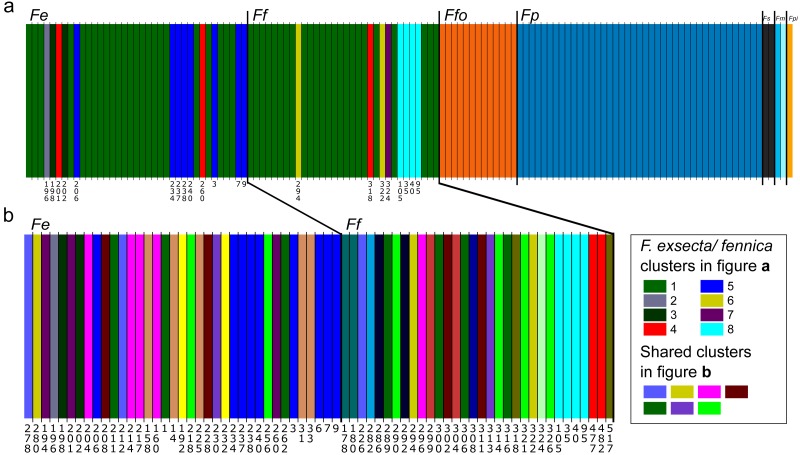
Bayesian clustering of seven *Coptoformica* species obtained with the software Baps. Abbreviations: Fe, *F. exsecta*; Ff, *F. fennica*; Ffo, *F. forsslundi*; Fp. *F. pressilabris*; Fs, *F. suecica*; Fm, *F. manchu*; Fpi, *F. pisarskii*. (A) Clustering for the whole dataset (the optimal *K* = 14). (B) Clustering for *F. exsecta* and *F. fennica* samples (the optimal *K* = 22).

In Bayesian clustering (Fig. 2A), the optimal number of genetic clusters for the whole dataset of one individual per nest was *K* = 14 (Posterior probability= 0.45), but other cluster numbers also gain large support (*K* = 13, *P* = 0.37; *K* = 12, *P* = 0.12; *K* = 15, *P* = 0.06). In the most optimal partition, *F. exsecta* and *F. fennica* share one major cluster, with additional smaller clusters, and the number of these additional *F. exsecta /fennica* clusters is the only difference between the other most optimal cluster numbers. Morphologically defined species represent all other clusters, and each species has only one cluster except *F. manchu* with its two samples clustering separately. When Bayesian clustering is repeated using only *F. exsecta* and *F. fennica* samples (Fig. 2B), the structure is broken down into several clusters of only few individuals, the most probable number of clusters being *K* = 22 (*P* = 0.38), *K* = 21 (*P* = 0.29), *K* = 20 (0.17) and *K* = 23 (0.14). In the most optimal partition, there are seven clusters shared between *F. exsecta* and *F. fennica* samples.

**Figure 3:**
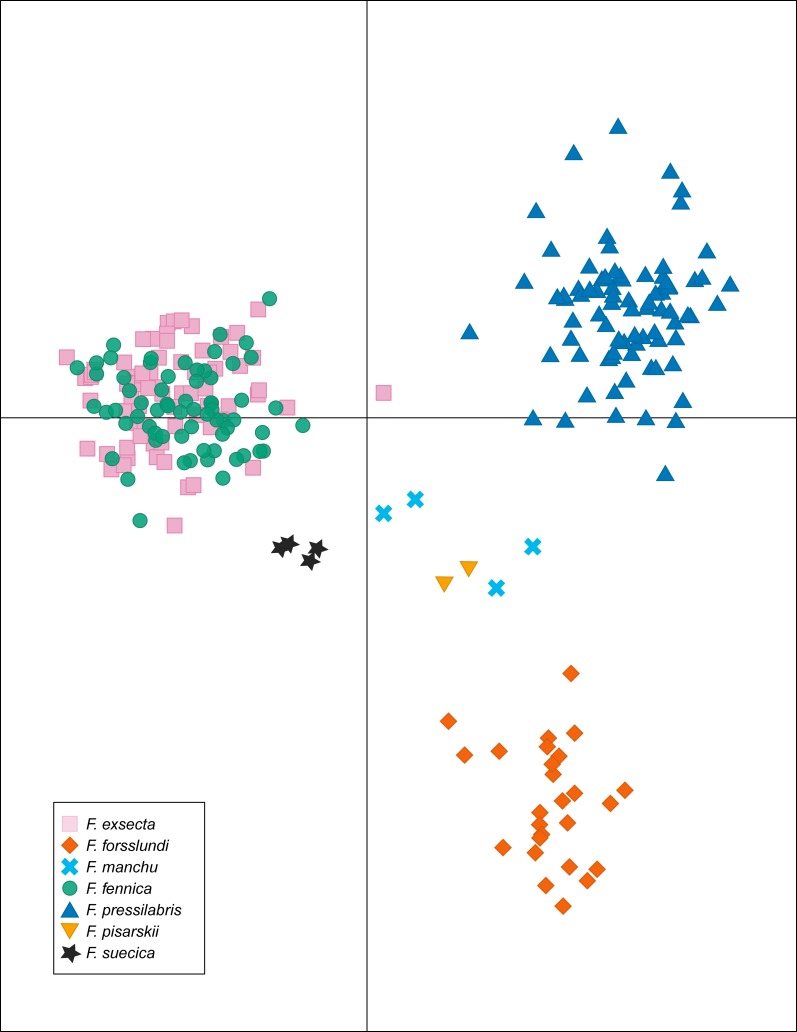
Discriminant analysis of principal components (DAPC) of the microsatellite data of seven *Coptoformica* species with 24 principal components included.

**Figure 4:**
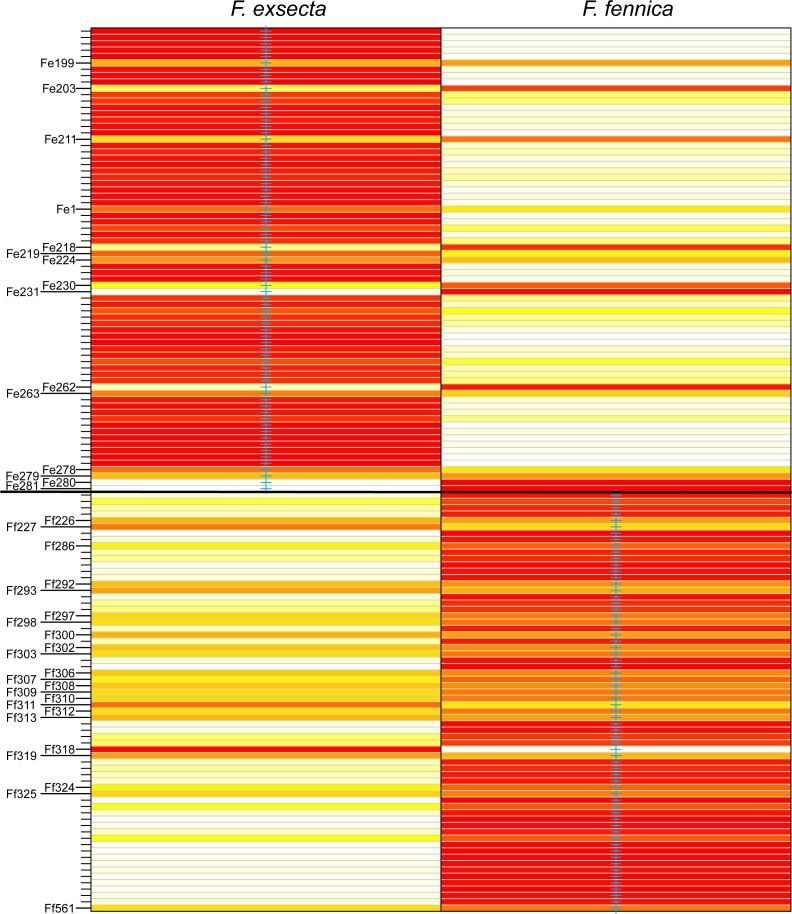
Assignment plot from discriminant analysis of principal components (DAPC). Blue cross marks the prior morphological species identity. Individuals are reassigned to these groups based on the DAPC model with 24 principal components. The color gradient represents membership probabilities (Red = 1 White = 0). Individuals that are assigned into the wrong group with >10% probability are named. Overall, the assignment of individuals to their morphological groups succeeds with the accuracy of 86.3% for *F. exsecta* and 86.4% for *F. fennica.*

In DAPC of the whole microsatellite dataset (Fig. 3), *F. exsecta* and *F. fennica* cluster together. Other morphologically defined species form more distinct clusters, clearly separated from other species, with the exception of the two individuals of *F. pisarskii* and four individuals of *F. manchu* grouping loosely together. The model’s ability to reassign individuals to their morphologically defined species is 100% for the other groups, but only 86.3% for *F. exsecta* and 86.4% for *F. fennica*, and in the cases when the assignment does not succeed according to morphology, *F. fennica* samples are always assigned to be *F. exsecta*, and vice versa. The consistency of DAPC classification with the morphological species identifications of *F. exsecta* and *F. fennica* samples is visualized in Fig. 4. When the model is fit for the subset of *F. exsecta* and *F. fennica* only, it is unable to reliably assign the samples to two groups with reasonably small number of PCs. The best possible fit is achieved with 63 PCs, over half of the total number of PCs, which makes the model overfitted. This means that the explanatory power of the model with additional samples would be very poor, as it already uses individual level characteristics instead of group level characteristics to separate the two groups. The overfitted model can assign all samples of *F. exsecta* correctly, but only 97% of *F. fennica* samples.

**Figure 5:**
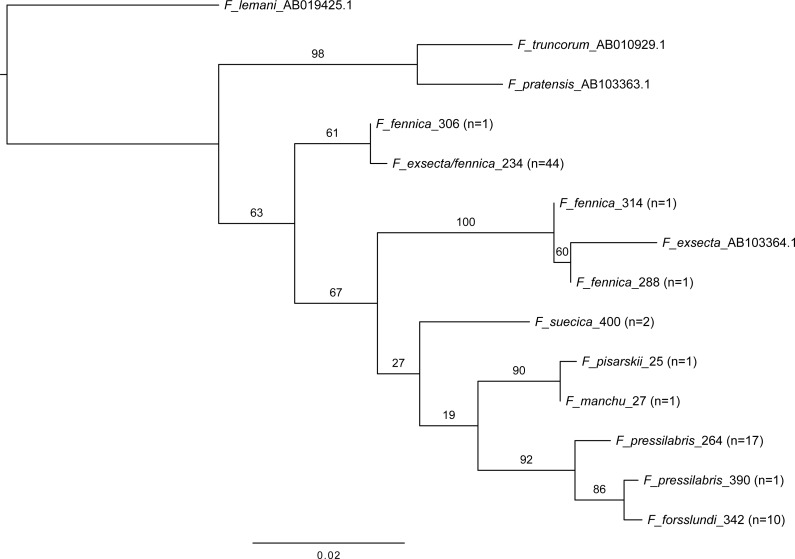
Maximum likelihood tree of COI barcodes of seven *Coptoformica* species and three additional *Formica* species. The additional samples obtained from GenBank. Bootstrap values shown next to the nodes. Note that some of the branches do not have sufficient bootstrap support and the tree should not be used to interpret the phylogeny.

In mitochondrial DNA barcoding, most studied species have species-specific haplotypes. *Formica pressilabris* has two haplotypes (diverging from each other by 6 nucleotides/1.14%), and *F. suecica* and *F. forsslundi* both only one. Also the single samples of *F. manchu* and *F. pisarskii* have unique haplotypes. *Formica exsecta* and *F. fennica* share the most common haplotype, which is also the only haplotype for *F. exsecta*. The *F. exsecta* haplotype from GenBank (AB103364.1) is different from the one obtained in this study. Apart from the shared *F. exsecta/fennica* haplotype, there are three additional haplotypes in *F. fennica* samples. The divergence among different *F. fennica* haplotypes varies between 1–18 nucleotides (0.19%–3.43%). The haplotype divergences between different species vary between 1–21 nucleotides (0.19%–4.00%). Table 3 shows all haplotype divergences measured in this study. The best scoring phylogenetic tree is presented in Fig. 5. The included *Coptoformica* samples form a clade. Two lineages composed of *F. fennica* and *F. exsecta* samples are recovered basal to the other *Coptoformica* species. *Formica fennica* and *F. exsecta* samples do not form monophyletic groups. In this taxon sampling *F. manchu* and F*. fennica* are not sister groups.

**Table 3:** Divergences between the COI barcode haplotypes found in this study, and one reference haplotype from Genbank (5). Below diagonal: number of differing nucleotides. Above diagonal: percentages of differing nucleotides.

**Haplotypes**	***n***	**1**	**2**	**3**	**4**	**5**	**6**	**7**	**8**	**9**	**10**	**11**
1 *F_fennica* _306	1		3.09	3.24	0.19	4.00	3.24	3.81	3.62	3.43	3.43	3.62
2 *F_fennica* _314	1	16		0.19	3.24	1.14	3.24	3.43	3.62	3.43	3.62	3.24
3 *F_fennica* _288	1	17	1		3.43	0.95	3.43	3.62	3.81	3.62	3.62	3.24
4 *F_exsecta/fennica* _234	44	1	17	18		3.81	3.43	4.00	3.81	3.62	3.62	3.81
5 F*_exsecta* _AB103364.1	–	21	6	5	20		4.00	3.81	4.00	3.81	3.81	3.81
6 *F_suecica* _400	2	17	17	18	18	21		2.48	2.29	3.24	3.24	3.62
7 *F_pisarskii* _25	1	20	18	19	21	20	13		0.19	2.48	2.48	2.86
8 *F_manchu* _27	1	19	19	20	20	21	12	1		2.29	2.29	2.67
9 *F_pressilabris* _264	17	18	18	19	19	20	17	13	12		1.14	1.14
10 *F_pressilabris* _390	1	18	19	19	19	20	17	13	12	6		0.38
11 *F_forsslundi* _342	10	19	17	17	20	20	19	15	14	6	2	

**Figure 6:**
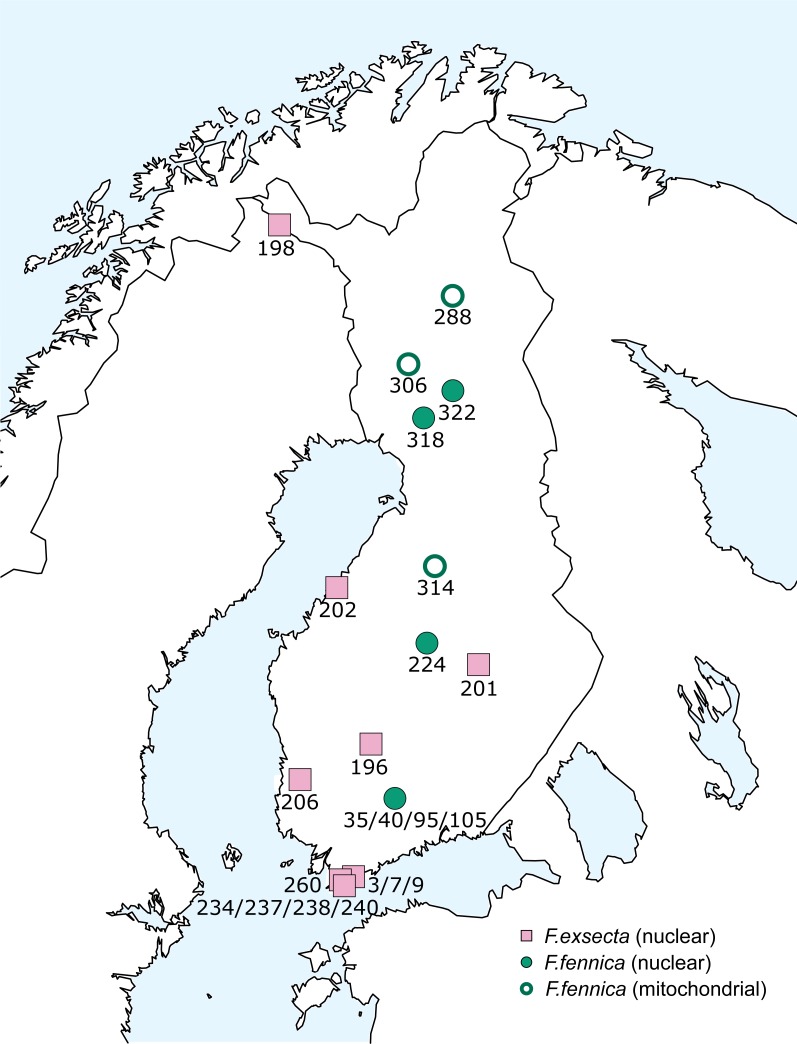
Geographical distribution of diverging *F. exsecta* and *F. fennica* samples. Individuals/populations that diverge from the main combined clusters of *F. exsecta/fennica* in nuclear or mitochondrial markers are shown. Nuclear clusters were determined with Baps from DNA microsatellite data and mitochondrial clusters as haplotypes of the partial COI barcode.

The geographical distribution of all individuals diverging from the main group of *F. exsecta/fennica* in either the nuclear or the mitochondrial dataset was mapped, but no clear patterns appear: the genetic differences are distributed throughout the sampling area (Fig. 6).

## Discussion

The morphological identifications for most of the *Coptoformica* species match the genetic patterns revealed in this study. Our results support the species identities of these species. In contrast, the morphological identifications of *F. exsecta* and *F. fennica* do not match the genetic patterns in any of the analyses. Both mitochondrial sequences and nuclear microsatellite genotypes reveal mixed patterns, with most of the samples of these two species clustering together regardless of morphology. One of the *F. fennica* populations sampled for this study was used in the original description of the species (Seifert, 2000). Also the samples from this population cluster together with *F. exsecta* samples in all analyses with both mitochondrial and nuclear markers. Thus, the hypothesis that the morphologically identified species *F. fennica* is also genetically differentiated, is not supported.

Genetic differentiation among individuals of *F. exsecta/fennica* group is distributed throughout the geographic range with no obviously distinct populations or areas. There is minor differentiation between *F. fennica* and *F. exsecta* samples overall, seen in the low but significant pairwise *F*_ST_ value calculated from microsatellite data. However, the significant effect of spatial distance, combined with the uneven sampling pattern, with more samples identified as *F. fennica* collected in northern areas of Finland and more samples identified as *F. exsecta* from southern areas, suggest this differentiation reflects isolation by distance rather than a species difference. Furthermore, the *F*_ST_ and Nei’s D values between samples of *F. exsecta* and *F. fennica* are overall drastically lower than the same values calculated between other pairs of species. This indicates an ongoing gene flow between morphologically identified *F. exsecta* and *F. fennica*. This is contrasted clearly with the other *Coptoformica* species that are more distinct genetic entities based on the *F*_ST_ values.

Reflecting the *F*_ST_ values, Bayesian clustering of the microsatellite data reveals clear structuring at the species level in *Coptoformica*, except within the *F. exsecta/fennica* group. Since the genetic differentiation between *F. exsecta* and *F. fennica* is minor, Baps analysis does not find stable partitions for this part of the data. The analysis does reveal some structuring, separating few individuals from both of these species into separate clusters. But since these individuals do not separate from the main group with other analysis methods, and the clusters correspond to geographical locations even when the geographic information was not used as informative prior, the structuring is most likely due to minor differentiation between local populations, and does not reflect species-level differences. The weakness of the structuring is shown in the low probability scores for the most optimal partitions, and in the way the structuring almost completely breaks down to the location level, when *F. exsecta/fennica* data are analyzed without the other species. It is also notable that in the analysis with only *F. exsecta* and *F. fennica,* several of the small clusters are shared between the two species, further affirming the mismatch between morphological species identification and molecular analysis. The other species cluster perfectly by morphological identifications.

*F. exsecta* and *F. fennica* cluster together also in discriminant analysis of principal components (DAPC) of the whole microsatellite dataset, and the model is unable to separate these groups reliably, although it does show minor genetic differentiation between some of the samples of *F. exsecta* and *F. fennica*. Since the analysis does cluster all of the other species with 100% accuracy, this result shows signs of admixture and misclassified individuals between *F. exsecta* and *F. fennica*. When *F. exsecta* and *F. fennica* samples are analyzed alone, the model with the highest accuracy and lowest error has to use over half of the total number of PCs, making it overfitted. Strikingly, even the overfitted model is unable to assign all of the samples of *F. fennica* correctly, which clearly shows that the species is not separated from *F. exsecta* but instead has individuals that are genetically extremely close to *F. exsecta*. Based on these results, we conclude that even though there is some differentiation between some of the samples of *F. exsecta* and *F. fennica*, the separation between these two species is substantially smaller than the separation between other species of the subgenus.

Also the mitochondrial data are clearly supporting the results of the nuclear data: most of *F. exsecta* and *F. fennica* samples share the same mitochondrial haplotype, whereas the other species have species-specific haplotypes. However, some *F. fennica* samples do diverge from this main haplotype. The pairwise sequence divergences between different *F. fennica* haplotypes vary from 0.19% to 3.43%. The average interspecific sequence divergence in *Coptoformica* has previously been reported to be 3.61% (Goropashnaya et al., 2012), although this number is based on longer sequences, a different mitochondrial gene and a larger geographic range that we used. Usually intraspecific diversity in COI barcodes is quite low, and sequence divergences of 2% or 3% have been suggested as suitable cut-off values for separating different species (Hebert et al., 2003; Smith, Fisher & Hebert, 2005). In a barcoding study of 51 ant species in Mauritius, a threshold of 2% sequence divergence was suitable (Smith & Fisher, 2009). However, Jansen, Savolainen & Vepsäläinen (2009) report intraspecific divergences up to 5.54% in Palearctic *Myrmica* species when sampling covers large geographic areas, and interspecific values as low as 0–0.96%. The latter is in line with the low values reported in this study between *F. pisarskii* and *F. manchu* (0.19%) and *F. pressilabris* and *F. forsslundi* (0.38%). Our data show that no arbitrary cut-off value should be trusted. Given the above, the mitochondrial sequence divergence in *F. fennica* samples in this study is within the bounds of intraspecific sequence divergence, but it is still on the high end of the scale, suggesting this group has genetic diversity worth additional studies.

Strikingly, when differences from the main haplotype occur in mitochondrial sequences of some *F. fennica* individuals, similar differentiation is not present in the nuclear DNA of these same individuals. Even though there is large variation in the *F. exsecta/fennica* group in both mitochondrial and nuclear datasets, no distinct sub-groups appear. Overall, the observed variation in *F. exsecta/fennica* group shows different patterns in mitochondrial and nuclear datasets, so that individuals divergent in one marker type belong to the major cluster in the other, with no geographical patterns to be seen (Fig. 6). Based on this result, all *F. exsecta/fennica* samples included in this study can be considered to be part of the same gene pool.

Mitochondrial DNA can differ from nuclear DNA due to various reasons, most notably incomplete lineage sorting, unrecognized paralogy, and hybridization resulting in introgression (Funk & Omland, 2003; Toews & Brelsford, 2012; Wallis et al., 2017). Similar types of patterns of mito-nuclear discordance are shown in hybrids of *Formica aquilonia* Yarrow, 1955 and *Formica polyctena* Förster, 1850 (Beresford et al., 2017). According to Funk & Omland (2003), if hybridization happened long ago, the persisting introgressed alleles are more likely to be phylogenetically basal and less likely to be geographically associated with the parental lineages. A historical hybridization of *F. exsecta* and a species not found in present-day Finland is a possible explanation for the observed non-monophyly and mito-nuclear discordance of *F. fennica* samples. In order to thoroughly investigate the observed nuclear and mitochondrial genetic variation in *F. exsecta/fennica* group, more extensive sampling at the population level would be needed.

The phylogenetic analysis presented in this study is based solely on partial COI data and a limited taxon sampling, which explains the low bootstrap support and the differences compared to the previously published partial phylogeny of *Coptoformica* species (Goropashnaya et al., 2012). Since the earlier phylogeny is based on substantially longer sequences and a better geographical coverage than used here, it should be considered more trustworthy. The main structure of the previous phylogeny with *F. exsecta* branching basally to the other *Coptoformica* species, is well supported also in the phylogeny presented here. The hypothesis that *F. fennica* samples form a distinct branch as a sister group with *F. manchu* was not supported. Although the sampling of this study is geographically restricted, and the data should not be used for full species delimitation nor for interpreting the exact phylogenetic relationships among these species, the result of *F. fennica* and *F. exsecta* grouping mostly together and branching basally to the other *Coptoformica* species is clear, and supports the results of nuclear data.

Our results show that the studied Finnish *F. fennica* populations should not be considered as a separate genetic entity from *F. exsecta*. None of the *F. fennica* populations were genetically differentiated from *F. exsecta* strongly enough to be considered a different species, including one of the populations used in the original description of *F. fennica* (Seifert, 2000). According to an earlier study, some of the samples that matched the species description of *F. fennica* actually belonged to the *rubens* morph of *F. exsecta* (Ødegaard, 2013). Based on this study, all Finnish *F. fennica* populations may also belong to the *rubens* (or some other) morph of *F. exsecta*. Since the sampling in this study does not cover the whole distribution area of *F. fennica*, the samples from other areas (i.e., Caucasus Seifert, 2000; Schultz & Seifert, 2007) and Norway (Suvák, 2013) should be re-analyzed in the light of these results for more accurate species delimitation.

Should some of the *F. exsecta* morphs represent different stages of a speciation continuum, it would be advisable to use an integrative approach combining both modern morphological and genetic methods, and possibly also other methods such as biochemical analyses (e.g.. cuticular hydrocarbons) and ethological and ecological analyses (Dayrat, 2005; Seifert, 2009; Schlick-Steiner et al., 2010). This would result in a fuller understanding of the observed diversity in *F. exsecta* and a more reliable species delimitation. Especially for conservation planning, it would be important to consider if some of the morphs of *F. exsecta* form evolutionarily significant units. Based on the data presented in this study, it is not possible to separate clear genetically distinct lineages, which has been an important criterion in many definitions of evolutionarily significant units (Fraser & Bernatchez, 2001). It is still worthwhile to consider whether the high morphological and genetic variation found in *F. exsecta* would be worth conserving. This study highlights the importance of taxonomic studies as reference for ecologists and conservation biologists.

## Conclusions

Both nuclear and mitochondrial markers fail to separate the species pair *F. exsecta* and *F. fennica* despite established, although not clear cut, morphological differences. The genetic variation within the *F. exsecta/fennica* group is extensive, but does not reflect the proposed morphological differences. It is impossible to divide these samples into two separate species based on our molecular data. The geographically restricted sampling of this study does not allow full species delimitation, but the result concerning the status of *F. fennica* is clear. Finnish *F. fennica* populations studied so far should not be considered a separate species, but merely a morph of *F. exsecta*.

##  Supplemental Information

10.7717/peerj.6013/supp-1 Supplemental Information 1 Sequences Raw data of the mitochondrial gene Cytochrome c oxidase subunit I sequences.

10.7717/peerj.6013/supp-2 Supplemental Information 2 Microsatellite data Raw data of the thirteen DNA microsatellite markers in genepop format.

10.7717/peerj.6013/supp-3 Supplemental Information 3 Sampling information

10.7717/peerj.6013/supp-4 Table S1 Laboratory protocols

10.7717/peerj.6013/supp-5 Table S2 Genetic variation in the 13 microsatellite loci studied

10.7717/peerj.6013/supp-6 Supplemental Information 4 Sequencing results of *Coptoformica* samples with the excluded samples

## Additional Information and Declarations

### DNA Deposition

The following information was supplied regarding the deposition of DNA sequences: The COI barcoding sequences were submitted to GenBank (and also included as a Supplemental File). GenBank accession numbers: BankIt2134696 F_fennica_306 MH649366, BankIt2134696 F_exsecta_fennica_234 MH649367. BankIt2134696 F_pressilabris_264 MH649368. BankIt2134696 F_pressilabris_390 MH649369. BankIt2134696 F_manchu_27 MH649370. BankIt2134696 F_pisarskii_25 MH649371. BankIt2134696 F_forsslundi_342 MH649372. BankIt2134696 F_suecica_400 MH649373. BankIt2134696 F_fennica_314 MH649374. BankIt2134696 F_fennica_288 MH649375.

### Data Availability

The following information was supplied regarding data availability: The sequence and microsatellite data are uploaded as Supplemental Files.
