# Supplementary material for: Genetic analysis reveals Finnish Formica fennica populations do not form a separate genetic entity from F. exsecta
Source: PeerJ. 2018 Dec 6;6:e6013. doi: 10.7717/peerj.6013 (PMC6286808; doi:10.7717/peerj.6013)
Supplement: Supplemental Information 3 [file peerj-06-6013-s003.docx]

**Supplement 1: Sampling information.**

**Supplement 1, Table 1: Samples included in this study.** The letters in IDs represent the morphology of samples. National Forest Inventory samples are specified with the abbreviation NFI in Location. The code (x.y) in column Location specify the nest (x) and individual (y). All samples were genotyped. Sequences were obtained from the samples marked with * on column Seq. Two dense *F. fennica* supercolonies have been intensely sampled for another study (FF_35-149 from Lammi and Ff_404-561 from Öby). Of these samples, 3-4 individuals per supercolony were included in the genotyping dataset and two individuals per supercolony in the sequencing dataset (**).

| **ID** | **Morph** | **Seq** | **Location** | **Year** | **Coordinates** | | **Identified by** |
| --- | --- | --- | --- | --- | --- | --- | --- |
| Fe_1 | *F. exsecta* | *** | Alkärr1.1 | 2010 | 60.06159 | 23.85701 | Väänänen, S. |
| Fe_2 | *F. exsecta* |  | Alkärr1.2 | 2010 | 60.06159 | 23.85701 | Väänänen, S. |
| Fe_3 | *F. exsecta* |  | Alkärr2.1 | 2010 | 60.06159 | 23.85701 | Väänänen, S. |
| Fe_4 | *F. exsecta* |  | Alkärr2.2 | 2010 | 60.06159 | 23.85701 | Väänänen, S. |
| Fe_5 | *F. exsecta* |  | Alkärr3.1 | 2010 | 60.06159 | 23.85701 | Väänänen, S. |
| Fe_6 | *F. exsecta* |  | Alkärr3.2 | 2010 | 60.06159 | 23.85701 | Väänänen, S. |
| Fe_7 | *F. exsecta* |  | Alkärr4.1 | 2010 | 60.06159 | 23.85701 | Väänänen, S. |
| Fe_8 | *F. exsecta* |  | Alkärr4.2 | 2010 | 60.06159 | 23.85701 | Väänänen, S. |
| Fe_9 | *F. exsecta* | *** | Alkärr5.1 | 2010 | 60.06159 | 23.85701 | Väänänen, S. |
| Fp_10 | *F. pressilabris* | *** | Antby1.1 | 2010 | 60.00741 | 23.68798 | Väänänen, S. |
| Fp_11 | *F. pressilabris* |  | Antby1.2 | 2010 | 60.00741 | 23.68798 | Väänänen, S. |
| Fp_12 | *F. pressilabris* |  | Antby2.1 | 2010 | 60.00741 | 23.68798 | Väänänen, S. |
| Fp_13 | *F. pressilabris* |  | Antby2.2 | 2010 | 60.00741 | 23.68798 | Väänänen, S. |
| Fe_14 | *F. exsecta* |  | Baggön1.1 | 2010 | 59.89272 | 23.51233 | Väänänen, S. |
| Fe_15 | *F. exsecta* | *** | Baggön1.2 | 2010 | 59.89272 | 23.51233 | Väänänen, S. |
| Fp_16 | *F. pressilabris* | *** | Baggön2.1 | 2010 | 59.89272 | 23.51233 | Väänänen, S. |
| Fp_17 | *F. pressilabris* |  | Baggön2.2 | 2010 | 59.89272 | 23.51233 | Väänänen, S. |
| Fp_18 | *F. pressilabris* |  | Baggön3.1 | 2010 | 59.89272 | 23.51233 | Väänänen, S. |
| Fp_19 | *F. pressilabris* |  | Baggön3.2 | 2010 | 59.89272 | 23.51233 | Väänänen, S. |
| Fp_20 | *F. pressilabris* |  | Baggön4.1 | 2010 | 59.89272 | 23.51233 | Väänänen, S. |
| Fp_21 | *F. pressilabris* |  | Baggön4.2 | 2010 | 59.89272 | 23.51233 | Väänänen, S. |
| Fpi_25 | *F. pisarskii* | *** | E49.1 | –––––––– Goropashanaya et al. 2012 –––––––– | | | |
| Fpi_26 | *F. pisarskii* |  | E49.2 |  |  |  |  |
| Fm_27 | *F. manchu* | *** | E51.1 |  |  |  |  |
| Fm_28 | *F. manchu* |  | E51.2 |  |  |  |  |
| Fm_29 | *F. manchu* |  | E52.1 |  |  |  |  |
| Fm_30 | *F. manchu* |  | E52.2 |  |  |  |  |
| Fe_31 | *F. exsecta* | *** | Furuskär 6.1 | 2013 | 59.83326 | 23.26555 | Sundström, L. |
| Fe_32 | *F. exsecta* |  | Furuskär 6.2 | 2013 | 59.83326 | 23.26555 | Sundström, L. |
| Fe_33 | *F. exsecta* |  | Furuskär 145B1 | 2013 | 59.83326 | 23.26555 | Sundström, L. |
| Fe_34 | *F. exsecta* |  | Furuskär 145B2 | 2013 | 59.83326 | 23.26555 | Sundström, L. |
| Ff_35-149 | *F. fennica* | **** | Supercolony in Lammi | 2008 | 61.06306 | 25.04979 | Punttila, P. |
| Fp_150 | *F. pressilabris* | *** | Finnpada1.1 | 2010 | 60.00881 | 23.87093 | Väänänen, S. |
| Fp_151 | *F. pressilabris* |  | Finnpada1.2 | 2010 | 60.00881 | 23.87093 | Väänänen, S. |
| Fp_152 | *F. pressilabris* | *** | Finnpada2.1 | 2010 | 60.00881 | 23.87093 | Väänänen, S. |
| Fp_153 | *F. pressilabris* |  | Finnpada2.2 | 2010 | 60.00881 | 23.87093 | Väänänen, S. |
| Fp_154 | *F. pressilabris* |  | Finnpada3.1 | 2010 | 60.00881 | 23.87093 | Väänänen, S. |
| Fp_155 | *F. pressilabris* |  | Finnpada3.2 | 2010 | 60.00881 | 23.87093 | Väänänen, S. |
| Fe_156 | *F. exsecta* | *** | Kaunissaari15.1 | 2013 | 60.17284 | 25.33687 | Punttila, P. |
| Fe_157 | *F. exsecta* |  | Kaunissaari15.2 | 2013 | 60.17284 | 25.33687 | Punttila, P. |
| Fe_158 | *F. exsecta* |  | Kaunissaari2.1 | 2013 | 60.17284 | 25.33687 | Punttila, P. |
| Fe_159 | *F. exsecta* |  | Kaunissaari2.2 | 2013 | 60.17284 | 25.33687 | Punttila, P. |
| Fe_160 | *F. exsecta* | *** | Kaunissaari31.1 | 2013 | 60.17284 | 25.33687 | Punttila, P. |
| Fe_161 | *F. exsecta* |  | Kaunissaari 31.2 | 2013 | 60.17284 | 25.33687 | Punttila, P. |
| Ff_178 | *F. fennica* | *** | Iisalmi1.1 | 2010 | 63.45991 | 27.18835 | Sorvari, J. |
| Ff_179 | *F. fennica* |  | Iisalmi1.2 | 2010 | 63.45991 | 27.18835 | Sorvari, J. |
| Ff_180 | *F. fennica* | *** | Iisalmi2.1 | 2010 | 63.45991 | 27.18835 | Sorvari, J. |
| Ff_181 | *F. fennica* |  | Iisalmi2.2 | 2010 | 63.45991 | 27.18835 | Sorvari, J. |
| Fe_192 | *F. exsecta* |  | Joskar 73.1 | 2013 | 59.84587 | 23.25544 | Sundström, L. |
| Fe_193 | *F. exsecta* |  | Joskar 73.2 | 2013 | 59.84587 | 23.25544 | Sundström, L. |
| Fe_194 | *F. exsecta* | *** | Orjansaari1.1 | 2013 | 61.22333 | 21.53373 | Sorvari, J. |
| Fe_195 | *F. exsecta* |  | Orjansaari1.2 | 2013 | 61.22333 | 21.53373 | Sorvari, J. |
| Fe_196 | *F. exsecta* | *** | Lakkasuo10.1 | 2015 | 61.78565 | 24.30634 | Sorvari, J. |
| Fe_197 | *F. exsecta* |  | Lakkasuo10.2 | 2015 | 61.78565 | 24.30634 | Sorvari, J. |
| Fe_198 | *F. exsecta* | *** | Ropinsalmi11.1 | 2010 | 68.68854 | 21.46448 | Sorvari, J. |
| Fe_199 | *F. exsecta* |  | Ropinsalmi11.2 | 2010 | 68.68854 | 21.46448 | Sorvari, J. |
| Fe_200 | *F. exsecta* | *** | Lippumäki12.1 | 2014 | 62.84179 | 27.64978 | Sorvari, J. |
| Fe_201 | *F. exsecta* |  | Lippumäki12.1 | 2014 | 62.84179 | 27.64978 | Sorvari, J. |
| Fe_202 | *F. exsecta* | *** | Finnilä2.1 | 2013 | 63.86572 | 23.24371 | Sorvari, J. |
| Fe_203 | *F. exsecta* |  | Finnilä2.2 | 2013 | 63.86572 | 23.24371 | Sorvari, J. |
| Fe_204 | *F. exsecta* | *** | Pahamaailma3.1 | 2011 | 65.43667 | 29.67361 | Sorvari, J. |
| Fe_205 | *F. exsecta* |  | Pahamaailma3.2 | 2011 | 65.43667 | 29.67361 | Sorvari, J. |
| Fe_206 | *F. exsecta* | *** | Sievari4.1 | 2012 | 61.31356 | 22.10003 | Sorvari, J. |
| Fe_207 | *F. exsecta* |  | Sievari4.2 | 2012 | 61.31356 | 22.10003 | Sorvari, J. |
| Fe_208 | *F. exsecta* | *** | Ruka5.1 | 2008 | 66.11887 | 29.11368 | Sorvari, J. |
| Fe_209 | *F. exsecta* |  | Ruka5.2 | 2008 | 66.11887 | 29.11368 | Sorvari, J. |
| Fe_210 | *F. exsecta* | *** | Kevo6.2 | 2008 | 69.74142 | 27.00087 | Sorvari, J. |
| Fe_211 | *F. exsecta* |  | Kevo6.2 | 2008 | 69.74142 | 27.00087 | Sorvari, J. |
| Fe_212 | *F. exsecta* | *** | Olostunturi7.1 | 2008 | 67.92681 | 23.80951 | Sorvari, J. |
| Fe_213 | *F. exsecta* |  | Olostunturi7.2 | 2008 | 67.92681 | 23.80951 | Sorvari, J. |
| Fe_214 | *F. exsecta* | *** | Sukeva8.1 | 2011 | 63.84628 | 27.44708 | Sorvari, J. |
| Fe_215 | *F. exsecta* |  | Sukeva8.2 | 2011 | 63.84628 | 27.44708 | Sorvari, J. |
| Fe_216 | *F. exsecta* | *** | Äkäskerontie9.1 | 2010 | 67.81971 | 24.06988 | Sorvari, J. |
| Fe_217 | *F. exsecta* |  | Äkäskerontie9.2 | 2010 | 67.81971 | 24.06988 | Sorvari, J. |
| Fe_218 | *F. exsecta* | *** | Kopparöfladan1.1 | 2010 | 59.90997 | 23.51818 | Väänänen, S. |
| Fe_219 | *F. exsecta* |  | Kopparöfladan1.2 | 2010 | 59.90997 | 23.51818 | Väänänen, S. |
| Fp_220 | *F. pressilabris* | *** | Kämpbacka1.1 | 2010 | 60.09954 | 24.18760 | Väänänen, S. |
| Fp_221 | *F. pressilabris* |  | Kämpbacka1.2 | 2010 | 60.09954 | 24.18760 | Väänänen, S. |
| Fp_222 | *F. pressilabris* |  | Kämpbacka2.1 | 2010 | 60.09954 | 24.18760 | Väänänen, S. |
| Fp_223 | *F. pressilabris* |  | Kämpbacka2.2 | 2010 | 60.09954 | 24.18760 | Väänänen, S. |
| Fe_224 | *F. exsecta* | *** | Kämpbacka3.1 | 2010 | 60.09954 | 24.18760 | Väänänen, S. |
| Fe_225 | *F. exsecta* |  | Kämpbacka3.2 | 2010 | 60.09954 | 24.18760 | Väänänen, S. |
| Ff_226 | *F. fennica* | *** | Iitto1.1 | 2010 | 68.72866 | 21.42183 | Sorvari, J. |
| Ff_227 | *F. fennica* |  | Iitto1.2 | 2010 | 68.72866 | 21.42183 | Sorvari, J. |
| Fe_228 | *F. exsecta* | *** | Längstrand1.1 | 2010 | 59.94770 | 23.17142 | Väänänen, S. |
| Fe_229 | *F. exsecta* |  | Längstrand1.2 | 2010 | 59.94770 | 23.17142 | Väänänen, S. |
| Fe_230 | *F. exsecta* |  | Mjölkholmen1.1 | 2010 | 60.03576 | 24.11302 | Väänänen, S. |
| Fe_231 | *F. exsecta* |  | Mjölkholmen1.2 | 2010 | 60.03576 | 24.11302 | Väänänen, S. |
| Fe_232 | *F. exsecta* | *** | Mjölkholmen2.1 | 2010 | 60.03576 | 24.11302 | Väänänen, S. |
| Fe_233 | *F. exsecta* |  | Mjölkholmen2.2 | 2010 | 60.03576 | 24.11302 | Väänänen, S. |
| Fe_234 | *F. exsecta* | *** | Prästkullantie1.1 | 2010 | 59.97957 | 23.35127 | Väänänen, S. |
| Fe_235 | *F. exsecta* |  | Prästkullantie1.2 | 2010 | 59.97957 | 23.35127 | Väänänen, S. |
| Fe_236 | *F. exsecta* |  | Prästkullantie2.1 | 2010 | 59.97957 | 23.35127 | Väänänen, S. |
| Fe_237 | *F. exsecta* |  | Prästkullantie2.2 | 2010 | 59.97957 | 23.35127 | Väänänen, S. |
| Fe_238 | *F. exsecta* |  | Prästkullantie3.1 | 2010 | 59.97957 | 23.35127 | Väänänen, S. |
| Fe_239 | *F. exsecta* |  | Prästkullantie3.2 | 2010 | 59.97957 | 23.35127 | Väänänen, S. |
| Fe_240 | *F. exsecta* |  | Prästkullantie4.1 | 2010 | 59.97957 | 23.35127 | Väänänen, S. |
| Fe_241 | *F. exsecta* |  | Prästkullantie4.2 | 2010 | 59.97957 | 23.35127 | Väänänen, S. |
| Fp_252 | *F. pressilabris* |  | Skaspåker1.1 | 2010 | 59.95213 | 23.66792 | Väänänen, S. |
| Fp_253 | *F. pressilabris* |  | Skaspåker1.2 | 2010 | 59.95213 | 23.66792 | Väänänen, S. |
| Fp_254 | *F. pressilabris* |  | Skaspåker2.1 | 2010 | 59.95213 | 23.66792 | Väänänen, S. |
| Fp_255 | *F. pressilabris* |  | Skaspåker2.2 | 2010 | 59.95213 | 23.66792 | Väänänen, S. |
| Fe_256 | *F. exsecta* |  | Skaspåker3.1 | 2010 | 59.95213 | 23.66792 | Väänänen, S. |
| Fe_257 | *F. exsecta* |  | Skaspåker3.2 | 2010 | 59.95213 | 23.66792 | Väänänen, S. |
| Fp_258 | *F. pressilabris* |  | Skaspåker4.1 | 2010 | 59.95213 | 23.66792 | Väänänen, S. |
| Fp_259 | *F. pressilabris* |  | Skaspåker4.2 | 2010 | 59.95213 | 23.66792 | Väänänen, S. |
| Fe_260 | *F. exsecta* |  | Skåldö1.1 | 2010 | 59.90116 | 23.47587 | Väänänen, S. |
| Fe_261 | *F. exsecta* | *** | Skåldö1.2 | 2010 | 59.90116 | 23.47587 | Väänänen, S. |
| Fe_262 | *F. exsecta* | *** | Skåldö2.1 | 2010 | 59.90116 | 23.47587 | Väänänen, S. |
| Fe_263 | *F. exsecta* |  | Skåldö2.2 | 2010 | 59.90116 | 23.47587 | Väänänen, S. |
| Fp_264 | *F. pressilabris* | *** | Storsand1.1 | 2010 | 59.94688 | 23.68753 | Väänänen, S. |
| Fp_265 | *F. pressilabris* |  | Storsand1.2 | 2010 | 59.94688 | 23.68753 | Väänänen, S. |
| Fp_266 | *F. pressilabris* |  | Storsand2.1 | 2010 | 59.94688 | 23.68754 | Väänänen, S. |
| Fp_267 | *F. pressilabris* |  | Storsand2.2 | 2010 | 59.94688 | 23.68754 | Väänänen, S. |
| Fp_268 | *F. pressilabris* |  | Storsand3.1 | 2010 | 59.94689 | 23.68754 | Väänänen, S. |
| Fp_269 | *F. pressilabris* |  | Storsand3.2 | 2010 | 59.94689 | 23.68754 | Väänänen, S. |
| Fp_270 | *F. pressilabris* |  | Särkkilen1.1 | 2010 | 59.95195 | 23.70879 | Väänänen, S. |
| Fp_271 | *F. pressilabris* | *** | Särkkilen1.2 | 2010 | 59.95195 | 23.70879 | Väänänen, S. |
| Fp_272 | *F. pressilabris* |  | Särkkilen2.1 | 2010 | 59.95195 | 23.70879 | Väänänen, S. |
| Fp_273 | *F. pressilabris* |  | Särkkilen2.2 | 2010 | 59.95195 | 23.70879 | Väänänen, S. |
| Fp_274 | *F. pressilabris* |  | Särkkilen3.1 | 2010 | 59.95195 | 23.70879 | Väänänen, S. |
| Fp_275 | *F. pressilabris* |  | Särkkilen3.2 | 2010 | 59.95195 | 23.70879 | Väänänen, S. |
| Fp_276 | *F. pressilabris* |  | Särkkilen4.1 | 2010 | 59.95195 | 23.70879 | Väänänen, S. |
| Fp_277 | *F. pressilabris* |  | Särkkilen4.2 | 2010 | 59.95195 | 23.70879 | Väänänen, S. |
| Fe_278 | *F. exsecta* | *** | NFIfe1.1 | 2005 | 63.50213 | 27.17526 | Punttila, P.; Sorvari, J. |
| Fe_279 | *F. exsecta* |  | NFIfe1.2 | 2005 | 63.50213 | 27.17526 | Punttila, P.; Sorvari, J. |
| Fe_280 | *F. exsecta* | *** | NFIfe10.1 | 2005 | 64.78043 | 26.86391 | Punttila, P.; Sorvari, J. |
| Fe_281 | *F. exsecta* |  | NFIfe10.2 | 2005 | 64.78043 | 26.86391 | Punttila, P.; Sorvari, J. |
| Ff_282 | *F. fennica* |  | NFIff11.1 | 2006 | 66.47080 | 29.06975 | Punttila, P. |
| Ff_283 | *F. fennica* |  | NFIff11.2 | 2006 | 66.47080 | 29.06975 | Punttila, P. |
| Ff_284 | *F. fennica* |  | NFIff12.1 | 2006 | 65.53854 | 25.36492 | Punttila, P. |
| Ff_285 | *F. fennica* |  | NFIff12.2 | 2006 | 65.53854 | 25.36492 | Punttila, P. |
| Ff_286 | *F. fennica* |  | NFIff13.1 | 2006 | 66.44964 | 23.69921 | Punttila, P. |
| Ff_287 | *F. fennica* | *** | NFIff13.2 | 2006 | 66.44964 | 23.69921 | Punttila, P. |
| Ff_288 | *F. fennica* | *** | NFIff14.1 | 2006 | 67.74515 | 26.84455 | Punttila, P. |
| Ff_289 | *F. fennica* |  | NFIff14.2 | 2006 | 67.74515 | 26.84455 | Punttila, P. |
| Ff_290 | *F. fennica* | *** | NFIff15.1 | 2006 | 68.09450 | 25.85298 | Punttila, P. |
| Ff_291 | *F. fennica* |  | NFIff15.2 | 2006 | 68.09450 | 25.85298 | Punttila, P. |
| Ff_292 | *F. fennica* |  | NFIff16.1 | 2006 | 64.27232 | 28.31113 | Punttila, P. |
| Ff_293 | *F. fennica* | *** | NFIff16.2 | 2006 | 64.27232 | 28.31113 | Punttila, P. |
| Ff_294 | *F. fennica* | *** | NFIff17.1 | 2007 | 64.41666 | 26.59394 | Punttila, P. |
| Ff_295 | *F. fennica* |  | NFIff17.2 | 2007 | 64.41666 | 26.59394 | Punttila, P. |
| Ff_296 | *F. fennica* | *** | NFIff18.1 | 2007 | 67.18054 | 24.07570 | Punttila, P. |
| Ff_297 | *F. fennica* |  | NFIff18.2 | 2007 | 67.18054 | 24.07570 | Punttila, P. |
| Ff_298 | *F. fennica* |  | NFIff19.1 | 2007 | 68.26434 | 24.88393 | Punttila, P. |
| Ff_299 | *F. fennica* |  | NFIff19.2 | 2007 | 68.26434 | 24.88393 | Punttila, P. |
| Ff_300 | *F. fennica* | *** | NFIff2.1 | 2005 | 68.27666 | 25.87288 | Punttila, P.; Sorvari, J. |
| Ff_301 | *F. fennica* |  | NFIff2.2 | 2005 | 68.27666 | 25.87288 | Punttila, P.; Sorvari, J. |
| Ff_302 | *F. fennica* | *** | NFIff20.1 | 2007 | 67.55174 | 28.68745 | Punttila, P. |
| Ff_303 | *F. fennica* |  | NFIff20.2 | 2007 | 67.55174 | 28.68745 | Punttila, P. |
| Ff_304 | *F. fennica* | *** | NFIff21.1 | 2007 | 65.03158 | 26.86900 | Punttila, P. |
| Ff_305 | *F. fennica* |  | NFIff21.2 | 2007 | 65.03158 | 26.86900 | Punttila, P. |
| Ff_306 | *F. fennica* |  | NFIff22.1 | 2008 | 66.83580 | 25.47023 | Punttila, P. |
| Ff_307 | *F. fennica* |  | NFIff22.2 | 2008 | 66.83580 | 25.47023 | Punttila, P. |
| Ff_308 | *F. fennica* | *** | NFIff23.1 | 2008 | 65.93811 | 28.60021 | Punttila, P. |
| Ff_309 | *F. fennica* |  | NFIff23.2 | 2008 | 65.93811 | 28.60021 | Punttila, P. |
| Ff_310 | *F. fennica* | *** | NFIff24.1 | 2008 | 67.54590 | 29.17738 | Punttila, P. |
| Ff_311 | *F. fennica* |  | NFIff24.2 | 2008 | 67.54590 | 29.17738 | Punttila, P. |
| Ff_312 | *F. fennica* | *** | NFIff25.1 | 2008 | 67.56025 | 27.29397 | Punttila, P. |
| Ff_313 | *F. fennica* |  | NFIff25.2 | 2008 | 67.56025 | 27.29397 | Punttila, P. |
| Ff_314 | *F. fennica* |  | NFIff26.1 | 2008 | 64.15088 | 26.29167 | Punttila, P. |
| Ff_315 | *F. fennica* |  | NFIff26.2 | 2008 | 64.15088 | 26.29167 | Punttila, P. |
| Ff_316 | *F. fennica* |  | NFIff27.1 | 2008 | 64.15088 | 26.29167 | Punttila, P. |
| Ff_317 | *F. fennica* |  | NFIff27.2 | 2008 | 64.15088 | 26.29167 | Punttila, P. |
| Ff_318 | *F. fennica* | *** | NFIff3.1 | 2005 | 66.12218 | 25.94274 | Punttila, P.; Sorvari, J. |
| Ff_319 | *F. fennica* |  | NFIff3.2 | 2005 | 66.12218 | 25.94274 | Punttila, P.; Sorvari, J. |
| Ff_320 | *F. fennica* | *** | NFIff4.1 | 2005 | 65.76697 | 26.82381 | Punttila, P.; Sorvari, J. |
| Ff_321 | *F. fennica* |  | NFIff4.2 | 2005 | 65.76697 | 26.82381 | Punttila, P.; Sorvari, J. |
| Ff_322 | *F. fennica* | *** | NFIff6.1 | 2005 | 66.48443 | 26.85243 | Punttila, P.; Sorvari, J. |
| Ff_323 | *F. fennica* |  | NFIff6.2 | 2005 | 66.48443 | 26.85243 | Punttila, P.; Sorvari, J. |
| Ff_324 | *F. fennica* | *** | NFIff7.1 | 2005 | 63.12761 | 26.04516 | Punttila, P.; Sorvari, J. |
| Ff_325 | *F. fennica* |  | NFIff7.2 | 2005 | 63.12761 | 26.04516 | Punttila, P.; Sorvari, J. |
| Ff_326 | *F. fennica* | *** | NFIff9.1 | 2005 | 65.76697 | 26.82381 | Punttila, P.; Sorvari, J. |
| Ff_327 | *F. fennica* |  | NFIff9.2 | 2005 | 65.76697 | 26.82381 | Punttila, P.; Sorvari, J. |
| Ffo_328 | *F. forsslundi* |  | NFIffo1.1 | 2005 | 64.65573 | 28.93459 | Punttila, P. |
| Ffo_329 | *F. forsslundi* | *** | NFIffo1.2 | 2005 | 64.65573 | 28.93459 | Punttila, P. |
| Ffo_330 | *F. forsslundi* | *** | NFIffo10.1 | 2008 | 63.62599 | 27.72862 | Punttila, P. |
| Ffo_331 | *F. forsslundi* |  | NFIffo10.2 | 2008 | 63.62599 | 27.72862 | Punttila, P. |
| Ffo_332 | *F. forsslundi* |  | NFIffo11.1 | 2008 | 64.38766 | 29.17486 | Punttila, P. |
| Ffo_333 | *F. forsslundi* |  | NFIffo11.2 | 2008 | 64.38766 | 29.17486 | Punttila, P. |
| Ffo_334 | *F. forsslundi* |  | NFIffo12.1 | 2008 | 64.38766 | 29.17486 | Punttila, P. |
| Ffo_335 | *F. forsslundi* |  | NFIffo12.2 | 2008 | 64.38766 | 29.17486 | Punttila, P. |
| Ffo_336 | *F. forsslundi* | *** | NFIffo13.1 | 2008 | 64.38766 | 29.17486 | Punttila, P. |
| Ffo_337 | *F. forsslundi* |  | NFIffo13.2 | 2008 | 64.38766 | 29.17486 | Punttila, P. |
| Ffo_338 | *F. forsslundi* | *** | NFIffo2.1 | 2005 | 65.16470 | 26.58253 | Punttila, P. |
| Ffo_339 | *F. forsslundi* |  | NFIffo2.2 | 2005 | 65.16470 | 26.58253 | Punttila, P. |
| Ffo_340 | *F. forsslundi* | *** | NFIffo3.1 | 2005 | 65.03094 | 27.45685 | Punttila, P. |
| Ffo_341 | *F. forsslundi* |  | NFIffo3.2 | 2005 | 65.03094 | 27.45685 | Punttila, P. |
| Ffo_342 | *F. forsslundi* | *** | NFIffo4.1 | 2005 | 62.87317 | 24.97645 | Punttila, P. |
| Ffo_343 | *F. forsslundi* |  | NFIffo4.2 | 2005 | 62.87317 | 24.97645 | Punttila, P. |
| Ffo_344 | *F. forsslundi* |  | NFIffo5.1 | 2005 | 62.87317 | 24.97645 | Punttila, P. |
| Ffo_345 | *F. forsslundi* |  | NFIffo5.2 | 2005 | 62.87317 | 24.97645 | Punttila, P. |
| Ffo_346 | *F. forsslundi* | *** | NFIffo6.1 | 2006 | 63.33328 | 22.97419 | Punttila, P. |
| Ffo_347 | *F. forsslundi* |  | NFIffo6.2 | 2006 | 63.33328 | 22.97419 | Punttila, P. |
| Ffo_348 | *F. forsslundi* |  | NFIffo7.1 | 2006 | 63.77532 | 28.30627 | Punttila, P. |
| Ffo_349 | *F. forsslundi* | *** | NFIffo7.2 | 2006 | 63.77532 | 28.30627 | Punttila, P. |
| Ffo_350 | *F. forsslundi* | *** | NFIffo8.1 | 2007 | 64.38381 | 24.27300 | Punttila, P. |
| Ffo_351 | *F. forsslundi* |  | NFIffo8.2 | 2007 | 64.38381 | 24.27300 | Punttila, P. |
| Ffo_352 | *F. forsslundi* | *** | NFIffo9.1 | 2008 | 64.77992 | 26.58849 | Punttila, P. |
| Ffo_353 | *F. forsslundi* |  | NFIffo9.2 | 2008 | 64.77992 | 26.58849 | Punttila, P. |
| Fp_354 | *F. pressilabris* | *** | NFIfp1.1 | 2005 | 61.68646 | 28.47034 | Punttila, P. |
| Fp_355 | *F. pressilabris* |  | NFIfp1.2 | 2005 | 61.68646 | 28.47034 | Punttila, P. |
| Fp_356 | *F. pressilabris* | *** | NFIfp10.1 | 2006 | 60.83189 | 25.56280 | Punttila, P. |
| Fp_357 | *F. pressilabris* |  | NFIfp10.2 | 2006 | 60.83189 | 25.56280 | Punttila, P. |
| Fp_358 | *F. pressilabris* | *** | NFIfp11.1 | 2006 | 63.13099 | 25.50182 | Punttila, P. |
| Fp_359 | *F. pressilabris* |  | NFIfp11.2 | 2006 | 63.13099 | 25.50182 | Punttila, P. |
| Fp_360 | *F. pressilabris* |  | NFIfp12.1 | 2007 | 62.86247 | 28.82155 | Punttila, P. |
| Fp_361 | *F. pressilabris* |  | NFIfp12.2 | 2007 | 62.86247 | 28.82155 | Punttila, P. |
| Fp_362 | *F. pressilabris* |  | NFIfp13.1 | 2007 | 63.52470 | 25.76260 | Punttila, P. |
| Fp_363 | *F. pressilabris* |  | NFIfp13.2 | 2007 | 63.52470 | 25.76260 | Punttila, P. |
| Fp_364 | *F. pressilabris* |  | NFIfp14.1 | 2007 | 62.32426 | 30.93358 | Punttila, P. |
| Fp_365 | *F. pressilabris* |  | NFIfp14.2 | 2007 | 62.32426 | 30.93358 | Punttila, P. |
| Fp_366 | *F. pressilabris* | *** | NFIfp15.1 | 2007 | 62.01957 | 26.88216 | Punttila, P. |
| Fp_367 | *F. pressilabris* |  | NFIfp15.2 | 2007 | 62.01957 | 26.88216 | Punttila, P. |
| Fp_368 | *F. pressilabris* |  | NFIfp16.1 | 2007 | 61.05156 | 26.44150 | Punttila, P. |
| Fp_369 | *F. pressilabris* |  | NFIfp16.2 | 2007 | 61.05156 | 26.44150 | Punttila, P. |
| Fp_370 | *F. pressilabris* |  | NFIfp17.1 | 2007 | 61.16261 | 27.10830 | Punttila, P. |
| Fp_371 | *F. pressilabris* |  | NFIfp17.2 | 2007 | 61.16261 | 27.10830 | Punttila, P. |
| Fp_372 | *F. pressilabris* | *** | NFIfp18.1 | 2008 | 61.03170 | 24.88759 | Punttila, P. |
| Fp_373 | *F. pressilabris* |  | NFIfp18.2 | 2008 | 61.03170 | 24.88759 | Punttila, P. |
| Fp_374 | *F. pressilabris* |  | NFIfp19.1 | 2008 | 60.73372 | 27.33138 | Punttila, P. |
| Fp_375 | *F. pressilabris* |  | NFIfp19.2 | 2008 | 60.73372 | 27.33138 | Punttila, P. |
| Fp_376 | *F. pressilabris* |  | NFIfp2.1 | 2005 | 62.63580 | 27.43718 | Punttila, P. |
| Fp_377 | *F. pressilabris* |  | NFIfp2.2 | 2005 | 62.63580 | 27.43718 | Punttila, P. |
| Fp_378 | *F. pressilabris* | *** | NFIfp20.1 | 2008 | 61.85143 | 29.80821 | Punttila, P. |
| Fp_379 | *F. pressilabris* |  | NFIfp20.2 | 2008 | 61.85143 | 29.80821 | Punttila, P. |
| Fp_380 | *F. pressilabris* | *** | NFIfp21.1 | 2008 | 64.01113 | 24.85152 | Punttila, P. |
| Fp_381 | *F. pressilabris* |  | NFIfp21.2 | 2008 | 64.01113 | 24.85152 | Punttila, P. |
| Fp_382 | *F. pressilabris* |  | NFIfp22.1 | 2008 | 64.77855 | 27.75953 | Punttila, P. |
| Fp_383 | *F. pressilabris* |  | NFIfp22.2 | 2008 | 64.77855 | 27.75953 | Punttila, P. |
| Fp_384 | *F. pressilabris* | *** | NFIfp3.1 | 2005 | 63.75267 | 27.46649 | Punttila, P. |
| Fp_385 | *F. pressilabris* |  | NFIfp3.2 | 2005 | 63.75267 | 27.46649 | Punttila, P. |
| Fp_386 | *F. pressilabris* |  | NFIfp4.1 | 2005 | 63.24237 | 24.64805 | Punttila, P. |
| Fp_387 | *F. pressilabris* | *** | NFIfp4.2 | 2005 | 63.24237 | 24.64805 | Punttila, P. |
| Fp_388 | *F. pressilabris* |  | NFIfp5.1 | 2005 | 61.48430 | 26.00207 | Punttila, P. |
| Fp_389 | *F. pressilabris* |  | NFIfp5.2 | 2005 | 61.48430 | 26.00207 | Punttila, P. |
| Fp_390 | *F. pressilabris* | *** | NFIfp6.1 | 2005 | 61.57547 | 28.91695 | Punttila, P. |
| Fp_391 | *F. pressilabris* |  | NFIfp6.2 | 2005 | 61.57547 | 28.91695 | Punttila, P. |
| Fp_392 | *F. pressilabris* |  | NFIfp7.1 | 2006 | 63.01259 | 27.44872 | Punttila, P. |
| Fp_393 | *F. pressilabris* |  | NFIfp7.2 | 2006 | 63.01259 | 27.44872 | Punttila, P. |
| Fp_394 | *F. pressilabris* | *** | NFIfp8.1 | 2006 | 61.59181 | 28.02322 | Punttila, P. |
| Fp_395 | *F. pressilabris* |  | NFIfp8.2 | 2006 | 61.59181 | 28.02322 | Punttila, P. |
| Fp_396 | *F. pressilabris* |  | NFIfp9.1 | 2006 | 61.59174 | 28.03263 | Punttila, P. |
| Fp_397 | *F. pressilabris* |  | NFIfp9.2 | 2006 | 61.59174 | 28.03263 | Punttila, P. |
| Fs_398 | *F. suecica* |  | NFIfs1.1 | 2005 | 62.60411 | 24.69871 | Punttila, P. |
| Fs_399 | *F. suecica* | *** | NFIfs1.2 | 2005 | 62.60411 | 24.69871 | Punttila, P. |
| Fs_400 | *F. suecica* | *** | NFIfs2.1 | 2005 | 62.60411 | 24.69871 | Punttila, P. |
| Fs_401 | *F. suecica* |  | NFIfs2.2 | 2005 | 62.60411 | 24.69871 | Punttila, P. |
| Ff_404-561 | *F. fennica^1^* | **** | Supercolony in Öby | 2011 | 59.93965 | 23.19798 | Punttila, P. |
| ^1^ Samples from this population that are not included in this study, have been inspected by B. Seifert, and his conclusion is that they present the *rubens* morph of *F. exsecta* (in litt. to H. Helanterä et al.). | | | | | | | |

**References:**

Goropashnaya. A. V. Fedorov. V. B.. Seifert. B.. & Pamilo. P. (2012). Phylogenetic relationships of Palaearctic *Formica* species (Hymenoptera. Formicidae) based on mitochondrial cytochrome B sequences. *PloS One*. **7**. e41697.
