# Supplementary material for: Genetic analysis reveals Finnish Formica fennica populations do not form a separate genetic entity from F. exsecta
Source: PeerJ. 2018 Dec 6;6:e6013. doi: 10.7717/peerj.6013 (PMC6286808; doi:10.7717/peerj.6013)
Supplement: Table S1 [file peerj-06-6013-s004.docx]

## Supplement 2: Laboratory protocols

#### DNA microsatellite genotyping

Fourteen DNA microsatellite loci were amplified with a forward primer labelled with fluorescent dye and a non-labelled reverse primer. Primers were pooled in four multiplex panels with 3-5 loci each. The primer dilutions were optimized for each panel based on the amplification results of preliminary analyses. Primer details for each panel are presented in Table 1. The primers were added to 5 μl of Type-it Microsatellite PCR solution (Qiagen) and 1 μl of undiluted sample DNA, and the reaction volume was adjusted to 10 μl with MilliQ water.

### The following PCR protocol was used for all panels: initial denaturation at 95˚C for 5 minutes; 29 cycles at 95˚C for 30 seconds, 57˚C for 90 seconds and 72˚C for 30 seconds; and final elongation at 60˚C for 30 minutes. PCR products were diluted 1/200 and analyzed in a 3730 ABI 3730 DNA analyzer using formamide as the buffer and GeneScan 500 ROX (Thermo Fisher Scientific) as the size standard.

#### mtDNA sequencing for DNA barcoding

Part of the COI gene was amplified with PCR primers designed by Seppä et al. (2011) using Phusion PCR kit (Finnzymes). The 20 μl reactions included 0.1 μl of forward and 0.1 μl of reverse primers (Oligomer. 100μM), 4 μl of Phusion HF Buffer (5x), 0.4 μl of dNTP (10mM), 0.2 μl Phusion High-Fidelity DNA Polymerase, 13.2 μl MilliQ water, and 2 μl of sample DNA. The DNA was undiluted for most of the samples but for some samples 1/5 dilution gave a better result. The following PCR protocol was used for most samples: initial denaturation at 98˚C for 30 seconds; 35 cycles at 98˚C for 10 seconds, 56˚C for 30 seconds and 72˚C for 30 seconds; and final elongation at 72˚C for 7 minutes. For poorly amplifying samples the program was lengthened with 5 additional cycles. PCR products were purified and sequenced 1-3 times per sample in the Institute of Biotechnology of University of Helsinki using the aforementioned primers (both forward and reverse).

**Supplement 2, Table 1.** DNA microsatellite primer details for the multiplex reactions.

| **Panel 1** |  |  | **Panel 2** |  |  |
| --- | --- | --- | --- | --- | --- |
| Primer | Amount* | Reference | Primer | Amount* | Reference |
| Fe51 (hex) | 0.015 μl | Gyllenstrand et al. 2002 | Fe16 (fam) | 0.03 μl | Gyllenstrand et al. 2002 |
| Fe13 (tamra) | 0.03 μl |  | Fe21 (hex) | 0.02 μl |  |
| Fe19 (fam) | 0.02 μl |  | Fy7 (tamra) | 0.02 μl | Hasegawa & Imai 2004 |
| Fe42 (hex) | 0.025 μl |  |  |  |  |
| Fy4 (fam) | 0.035 μl | Hasegawa & Imai 2004 |  |  |  |
| **Panel 3** |  |  | **Panel 4** |  |  |
| Primer | Amount* | Reference | Primer | Amount* | Reference |
| Fe11 (tamra) | 0.015 μl | Gyllenstrand et al. 2002 | Fe37 (fam) | 0.04 μl | Gyllenstrand et al. 2002 |
| Fe38 (hex) | 0.015 μl |  | ** Fe17 (hex) | 0.04 μl |  |
| Fl21 (fam) | 0.04 μl | Chapuisat 1996 | P22 (tamra) | 0.005 μl | Trontti et al. 2003 |
| * The mentioned amounts of both forward and reverse primer stock solutions (Oligomer. 100μM) were used in each reaction. | | | | | |
| ** Locus was removed from the dataset after the initial analysis due to poor amplification in *F. forsslundi.* | | | | | |

**References:**

Chapuisat. M. (1996). Characterization of microsatellite loci in Formica lugubris B and their variability in other ant species. Molecular Ecology. 5. 599–601.

Gyllenstrand. N.. Gertsch. P. J.. & Pamilo. P. (2002). Polymorphic microsatellite DNA markers in the ant Formica exsecta. Molecular Ecology Notes. 2. 67–69.

Hasegawa. E.. & Imai. S. (2004). Characterization of microsatellite loci in red wood ants Formica (s. str.) spp. and the related genus Polyergus. Molecular Ecology Notes. 4. 200–203.

Trontti. K.. Tay. W. T.. & Sundström. L. (2003). Polymorphic microsatellite markers for the ant Plagiolepis pygmaea. Molecular Ecology Notes. 3. 575–577.

Seppä. P.. Helanterä. H.. Trontti. K.. Punttila. P.. Chernenko. A.. Martin. S. J.. & Sundström. L. (2011). The many ways to delimit species: hairs. genes and surface chemistry. *Myrmecological News*. **15**. 31–41.
