# Supplementary material for: Genetic analysis reveals Finnish Formica fennica populations do not form a separate genetic entity from F. exsecta
Source: PeerJ. 2018 Dec 6;6:e6013. doi: 10.7717/peerj.6013 (PMC6286808; doi:10.7717/peerj.6013)
Supplement: Table S2 [file peerj-06-6013-s005.docx]

**Supplement 3, Table 1. Genetic variation in the 13 microsatellite loci studied.**

|  | ***F. exsecta* (n=69)** | | | | |  | ***F. fennica* (n=63)** | | | | |
| --- | --- | --- | --- | --- | --- | --- | --- | --- | --- | --- | --- |
| **Locus** | **N** | **Na** | **Npa** | ***H*_E_** | **A_R_** |  | **N** | **Na** | **Npa** | ***H*_E_** | **A_R_** |
| **Fe11** | 68 | 5 | 0 | 0.64 | 5.2 |  | 63 | 5 | 0 | 0.66 | 4.8 |
| **Fe13** | 68 | 13 | 0 | 0.89 | 12.4 |  | 62 | 13 | 1 | 0.88 | 12.7 |
| **Fe16** | 69 | 24 | 3 | 0.93 | 18.6 |  | 63 | 24 | 3 | 0.93 | 18.8 |
| **Fe19** | 69 | 4 | 0 | 0.38 | 3.5 |  | 63 | 5 | 0 | 0.70 | 5.0 |
| **Fe21** | 69 | 5 | 1 | 0.62 | 4.5 |  | 63 | 5 | 1 | 0.54 | 4.1 |
| **Fe37** | 69 | 9 | 0 | 0.80 | 8.2 |  | 63 | 10 | 1 | 0.77 | 8.7 |
| **Fe38** | 68 | 37 | 14 | 0.95 | 25.0 |  | 63 | 35 | 14 | 0.95 | 24.1 |
| **Fe42** | 68 | 6 | 0 | 0.77 | 6.4 |  | 60 | 6 | 0 | 0.78 | 6.7 |
| **Fe51** | 68 | 9 | 2 | 0.60 | 8.8 |  | 63 | 8 | 1 | 0.63 | 6.5 |
| **Fl21** | 68 | 24 | 10 | 0.91 | 19.0 |  | 57 | 21 | 4 | 0.92 | 18.2 |
| **Fy4** | 69 | 4 | 0 | 0.65 | 3.4 |  | 57 | 4 | 0 | 0.67 | 4.4 |
| **Fy7** | 69 | 8 | 2 | 0.70 | 6.9 |  | 61 | 10 | 1 | 0.78 | 8.6 |
| **P22** | 68 | 10 | 1 | 0.80 | 8.8 |  | 63 | 10 | 1 | 0.78 | 8.5 |
|  |  |  |  |  |  |  |  |  |  |  |  |
|  | ***F. forsslundi* (n=26)** | | | | |  | ***F. pressilabris* (n=79)** | | | | |
| **Locus** | **N** | **Na** | **Npa** | ***H*_E_** | **A_R_** |  | **N** | **Na** | **Npa** | ***H*_E_** | **A_R_** |
| **Fe11** | 26 | 3 | 0 | 0.53 | 3.0 |  | 79 | 6 | 2 | 0.67 | 5.1 |
| **Fe13** | 25 | 6 | 4 | 0.75 | 7.0 |  | 78 | 13 | 5 | 0.86 | 11.1 |
| **Fe16** | 26 | 14 | 7 | 0.85 | 14.0 |  | 79 | 18 | 2 | 0.89 | 13.2 |
| **Fe19** | 26 | 7 | 4 | 0.71 | 7.0 |  | 79 | 7 | 3 | 0.60 | 5.4 |
| **Fe21** | 26 | 2 | 0 | 0.49 | 2.0 |  | 79 | 6 | 2 | 0.72 | 4.7 |
| **Fe37** | 26 | 5 | 1 | 0.52 | 5.0 |  | 78 | 11 | 3 | 0.78 | 9.0 |
| **Fe38** | 26 | 7 | 0 | 0.77 | 7.0 |  | 79 | 13 | 0 | 0.83 | 9.4 |
| **Fe42** | 25 | 1 | 0 | 0.00 | 2.0 |  | 79 | 5 | 0 | 0.53 | 3.6 |
| **Fe51** | 26 | 4 | 0 | 0.46 | 4.0 |  | 79 | 7 | 2 | 0.75 | 5.8 |
| **Fl21** | 26 | 9 | 0 | 0.80 | 9.0 |  | 78 | 5 | 2 | 0.70 | 5.3 |
| **Fy4** | 24 | 4 | 1 | 0.66 | 5.0 |  | 74 | 3 | 0 | 0.60 | 4.0 |
| **Fy7** | 24 | 5 | 1 | 0.55 | 6.0 |  | 76 | 13 | 7 | 0.81 | 10.7 |
| **P22** | 26 | 3 | 0 | 0.54 | 3.0 |  | 77 | 6 | 0 | 0.56 | 5.5 |

n=sample size (number of individuals). N=sample size per locus (N < n indicates missing data). Na=number of alleles. Npa=number of private alleles. *H*_E_=expected heterozygosity. A_R_=allelic richness
