# Supplementary material for: Genetic analysis reveals Finnish Formica fennica populations do not form a separate genetic entity from F. exsecta
Source: PeerJ. 2018 Dec 6;6:e6013. doi: 10.7717/peerj.6013 (PMC6286808; doi:10.7717/peerj.6013)
Supplement: Supplemental Information 4 [file peerj-06-6013-s006.docx]

**Suplement 4: Sequencing results of *Coptoformica* samples also containing the excluded samples**

**Supplement 4, figure 1. Maximum likelihood tree of COI barcodes of seven *Coptoformica* species and three additional *Formica* species**.

Bootstrap values shown next to the nodes. The haplotypes *F_fennica*_312, *F_fennica*_304 and *F_ fennica*_310 do not seem to belong to the otherwise monophyletic *Coptoformica*, and were therefore excluded from the final analysis.


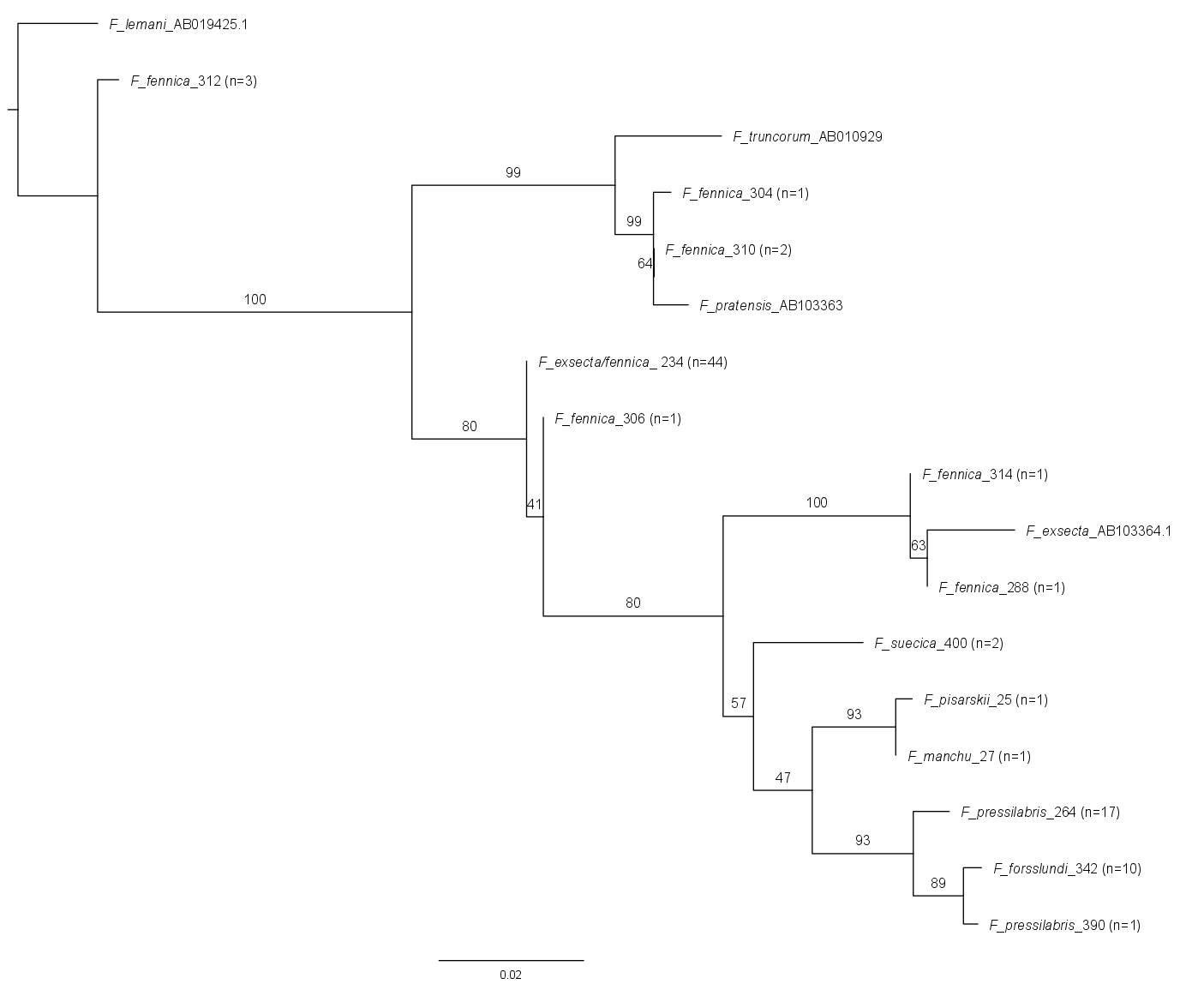


**Supplement 4, Table 1. Divergences between all of the COI barcode haplotypes found in this study.**

Below diagonal: number of differing nucleotides. Above diagonal: percentages of differing nucleotides.

| Haplotypes: | n | 1 | 2 | 3 | 4 | 5 | 6 | 7 | 8 | 9 | 10 | 11 | 12 | 13 | 14 |
| --- | --- | --- | --- | --- | --- | --- | --- | --- | --- | --- | --- | --- | --- | --- | --- |
| 1 *F_fennica*_312 | 3 |  | 4.57 | 4.57 | 4.19 | 4.95 | 4.95 | 4.00 | 5.33 | 5.71 | 5.14 | 4.95 | 5.52 | 4.76 | 4.95 |
| 2 *F_fennic*a_310 | 2 | 24 |  | 0.19 | 3.43 | 4.57 | 4.76 | 3.24 | 5.14 | 5.14 | 5.33 | 5.14 | 4.76 | 4.38 | 4.57 |
| 3 *F_fennica*_304 | 1 | 24 | 1 |  | 3.43 | 4.57 | 4.76 | 3.24 | 5.14 | 5.14 | 5.33 | 5.14 | 4.76 | 4.38 | 4.57 |
| 4 *F_fennica*_306 | 1 | 22 | 18 | 18 |  | 3.05 | 3.24 | 0.19 | 4.00 | 3.24 | 3.81 | 3.62 | 3.43 | 3.43 | 3.62 |
| 5 *F_fennica*_314 | 1 | 26 | 24 | 24 | 16 |  | 0.19 | 3.24 | 1.14 | 3.24 | 3.43 | 3.62 | 3.43 | 3.62 | 3.24 |
| 6 *F_fennica*_288 | 1 | 26 | 25 | 25 | 17 | 1 |  | 3.43 | 0.95 | 3.43 | 3.62 | 3.81 | 3.62 | 3.62 | 3.24 |
| 7 *F_exsect*a*/fennica*_234 | 44 | 21 | 17 | 17 | 1 | 17 | 18 |  | 3.81 | 3.43 | 4.00 | 3.81 | 3.62 | 3.62 | 3.81 |
| 8 *F_exsecta*_AB103364.1 | - | 28 | 27 | 27 | 21 | 6 | 5 | 20 |  | 4 | 3.81 | 4.00 | 3.81 | 3.81 | 3.81 |
| 9 *F_suecic*a_400 | 2 | 30 | 27 | 27 | 17 | 17 | 18 | 18 | 21 |  | 2.48 | 2.29 | 3.24 | 3.24 | 3.62 |
| 10 *F_pisarskii*_25 | 1 | 27 | 28 | 28 | 20 | 18 | 19 | 21 | 20 | 13 |  | 0.19 | 2.48 | 2.48 | 2.86 |
| 11 *F_manchu*_27 | 1 | 26 | 27 | 27 | 19 | 19 | 20 | 20 | 21 | 12 | 1 |  | 2.29 | 2.29 | 2.67 |
| 12 *F_pressilabris*_264 | 17 | 29 | 25 | 25 | 18 | 18 | 19 | 19 | 20 | 17 | 13 | 12 |  | 1.14 | 1.14 |
| 13 *F_pressilabris*_390 | 1 | 25 | 23 | 23 | 18 | 19 | 19 | 19 | 20 | 17 | 13 | 12 | 6 |  | 0.38 |
| 14 *F_forsslundi*_342 | 10 | 26 | 24 | 24 | 19 | 17 | 17 | 20 | 20 | 19 | 15 | 14 | 6 | 2 |  |
